# Prevalence of Carbapenem-Resistant Gram-Negative Bacilli from Intensive Care Units from Latin America and the Caribbean: A Systematic Review and Meta-Analysis

**DOI:** 10.3390/antibiotics15020209

**Published:** 2026-02-14

**Authors:** Jesús D. Rojas, Mercy Carolina Merejildo Vera, Juan Carlos Benites Azabache, Valeria De La Cruz Surco, Juan Raúl Lucas López, Rafael Pichardo-Rodriguez

**Affiliations:** 1 Programa Académico de Tecnología Médica en Laboratorio Clínico y Anatomía Patológica, Facultad de Ciencias de la Salud, Universidad Privada Norbert Wiener, Lima 15046, Peru; 2 School of Veterinary Medicine, Universidad Nacional Mayor de San Marcos, Lima 15021, Peru; jrlucas.pe@gmail.com; 3 Instituto de Investigaciones en Ciencias Biomédicas (INICIB), Universidad Ricardo Palma, Lima 15039, Peru; 4 Hospital de Apoyo II-2 de Sullana, Sullana 20103, Peru; 5 Grupo Peruano de Evidencia Clínica y Real-World Evidence (RpeCRWD), Sullana 20101, Peru

**Keywords:** carbapenems, drug resistance, bacterial, Gram-negative bacteria, intensive care units, Latin America, Caribbean region, prevalence, systematic review, meta-analysis

## Abstract

**Background:** Carbapenem-resistant Gram-negative bacilli (CR-GNB) represent a critical threat to patients in intensive care units (ICUs), where limited therapeutic options contribute to elevated mortality. In Latin America and the Caribbean (LAC), the epidemiological burden of CR-GNB remains insufficiently characterized due to fragmented surveillance systems. This systematic review aimed to synthesize pooled prevalence estimates of CR-GNB among Gram-negative isolates recovered from ICUs across LAC countries. **Methods:** This systematic review was prospectively registered with PROSPERO (CRD420251177826), followed PRISMA 2020 guidelines and the JBI Manual for prevalence reviews. We searched PubMed, Scopus, LILACS, and SciELO from January 2015 to October 2025 without language restrictions. Observational studies reporting phenotypic carbapenem resistance data from ICUs in LAC countries were eligible. Two reviewers independently screened studies and extracted data. A two-level multilevel generalized linear mixed model (GLMM) with logit transformation was employed using a random-effects approach. Risk of bias was assessed using the JBI critical appraisal checklist. **Results:** Of 25 eligible studies spanning eight countries, 12 contributed 49 observations to quantitative synthesis. Overall pooled prevalence of CR-GNB was 28.88% (95% CI: 17.32–44.05%), with considerable heterogeneity (I^2^ = 95.24%). Species-specific prevalence was highest for *Acinetobacter baumannii* (72.58%), followed by *Klebsiella pneumoniae* (37.48%) and *Pseudomonas aeruginosa* (29.93%). Regional stratification revealed higher prevalence in South America (31.81%) compared to North America (22.65%) and the Caribbean (11.63%). **Conclusions:** Nearly one-third of Gram-negative isolates from LAC ICUs exhibit carbapenem resistance, with *A. baumannii* predominating. Substantial inter-study heterogeneity underscores the need for standardized regional surveillance networks and coordinated antimicrobial stewardship initiatives.

## 1. Introduction

Antimicrobial resistance (AMR) represents one of the most formidable challenges confronting global public health in the twenty-first century. In 2019, bacterial AMR was directly responsible for an estimated 1.27 million deaths worldwide, with an additional 4.95 million deaths associated with resistant infections [1]. The World Health Organization (WHO) has designated carbapenem-resistant Gram-negative bacilli (CR-GNB) among its critical priority pathogens, recognizing the urgent need for novel therapeutic agents and containment strategies [2,3]. This classification encompasses carbapenem-resistant *Enterobacterales* (CRE), *Acinetobacter baumannii* (CR-Ab), and *Pseudomonas aeruginosa* (CR-Pa)—organisms that collectively account for a substantial proportion of healthcare-associated infections globally [4]. Surveillance data from the European Antimicrobial Resistance Surveillance Network (EARS-Net) and the Global Antimicrobial Resistance and Use Surveillance System (GLASS) demonstrate alarming resistance trends, with carbapenem resistance exceeding 50% in certain pathogens across multiple geographic regions [5,6].

Intensive care units (ICUs) constitute epidemiological epicenters for the emergence and dissemination of CR-GNB. The convergence of multiple risk factors—including widespread utilization of invasive devices (mechanical ventilation, central venous catheters, urinary catheters), prolonged hospitalization, immunocompromised patient populations, and extensive exposure to broad-spectrum antimicrobials—creates an environment conducive to the selection and transmission of resistant organisms [7,8]. Critically ill patients harboring CR-GNB infections face substantially elevated mortality, with reported rates ranging from 27% to 52% depending on the pathogen and infection site [9,10]. Beyond clinical outcomes, these infections impose considerable economic burdens through extended hospital stays, increased resource utilization, and requirements for expensive last-resort antimicrobial agents [11].

The molecular epidemiology of carbapenem resistance exhibits marked geographic heterogeneity driven by distinct carbapenemase enzyme families. *Klebsiella pneumoniae* carbapenemase (KPC) predominates in the Americas, while New Delhi metallo-β-lactamase (NDM) has achieved widespread dissemination across the Indian subcontinent and beyond [12,13]. The OXA-48-like enzymes demonstrate particular prevalence in the Mediterranean basin, whereas Verona integron-encoded metallo-β-lactamase (VIM) and Imipenemase (active on imipenem metallo-β-lactamase) (IMP) exhibit regional clusters [14]. Horizontal gene transfer facilitated by mobile genetic elements—particularly conjugative plasmids and transposons—enables rapid inter-species and inter-institutional dissemination, accelerating the global spread of these resistance determinants [15].

Latin America and the Caribbean (LAC) represent a region of particular concern regarding CR-GNB epidemiology. Healthcare system fragmentation, variable diagnostic capacity, poor healthcare infrastructures, and heterogeneous antimicrobial stewardship implementation create conditions favorable for resistance emergence [16]. Although the Latin American Network for Antimicrobial Resistance Surveillance (ReLAVRA+) has substantially strengthened regional surveillance capacity since 1996, coverage and reporting remain inconsistent across member nations [17]. Available evidence indicates that KPC-producing *Enterobacterales* have achieved endemic status throughout much of South America, with NDM-producers demonstrating increasing prevalence in several countries [18,19]. The COVID-19 pandemic further exacerbated regional AMR challenges through disruption of stewardship programs, increased empirical antimicrobial use, and diversion of infection prevention resources [20,21]. The COVID-19 pandemic significantly amplified regional AMR challenges through multiple converging mechanisms. During the pandemic’s early phases, empirical antibiotic prescribing surged, with documented increases in carbapenem, ceftriaxone, and azithromycin consumption across LAC countries, despite bacterial co-infection rates of only 6.9% among hospitalized COVID-19 patients [22]. ReLAVRA reported that during 2020–2021, multiple LAC countries experienced clinical emergence of carbapenemase-producing Enterobacterales not previously characterized locally, increased prevalence of existing carbapenemases, and unprecedented co-production of multiple carbapenemases in individual isolates [20]. Surveillance data from Brazilian ICUs demonstrated that carbapenem resistance increased during the surge period and, although decreasing post-surge, remained higher than pre-pandemic levels [23]. Concurrently, infection prevention resources were diverted toward aerosol transmission control, often compromising contact precautions essential for multidrug-resistant organism containment. Antimicrobial stewardship programs experienced substantial disruption, with reduced oversight of prescribing practices and delayed the implementation of de-escalation strategies [24]. The Antimicrobial Testing Leadership and Surveillance (ATLAS) surveillance program (2018–2022) confirmed that CR-GNB rates in LAC were consistently higher than in North America and Europe, with prevalence rising further during the pandemic, with CRAB often exceeding 50% and CRE surpassing 10% [25]. These pandemic-associated factors created conditions favorable for resistance amplification, with potential long-term implications for endemic resistance baselines in the region.

Despite the recognized clinical significance of CR-GNB in LAC critical care settings, pooled prevalence estimates specific to ICU populations remain absent from the literature. Existing systematic reviews have predominantly focused on global or high-income country perspectives, inadequately representing the distinct epidemiological context of LAC nations [26]. Furthermore, species-specific prevalence data and the regional distribution of carbapenemase genes have not been systematically synthesized, limiting the evidence base available for informed policy development and resource allocation.

This systematic review and meta-analysis aims to address these knowledge gaps by synthesizing the prevalence of CR-GNB among Gram-negative isolates from ICU patients across LAC. Secondary objectives include stratification of prevalence by bacterial species (*K. pneumoniae*, *A. baumannii*, *Pseudomonas aeruginosa*, *Enterobacter clocacae* complex, and *Escherichia coli*), geographic subregion, and molecular epidemiology where data permit. This protocol was prospectively registered in PROSPERO (CRD420251177826).

## 2. Results

### 2.1. Study Selection

The systematic literature search across four electronic databases yielded 595 records: PubMed (*n* = 190), Scopus (*n* = 260), LILACS (*n* = 61), and SciELO (*n* = 84). Following the removal of 218 duplicate records, 377 unique citations underwent title and abstract screening. Of these, 209 were excluded based on predefined criteria, leaving 168 articles for full-text eligibility assessment.

**Figure 1:**
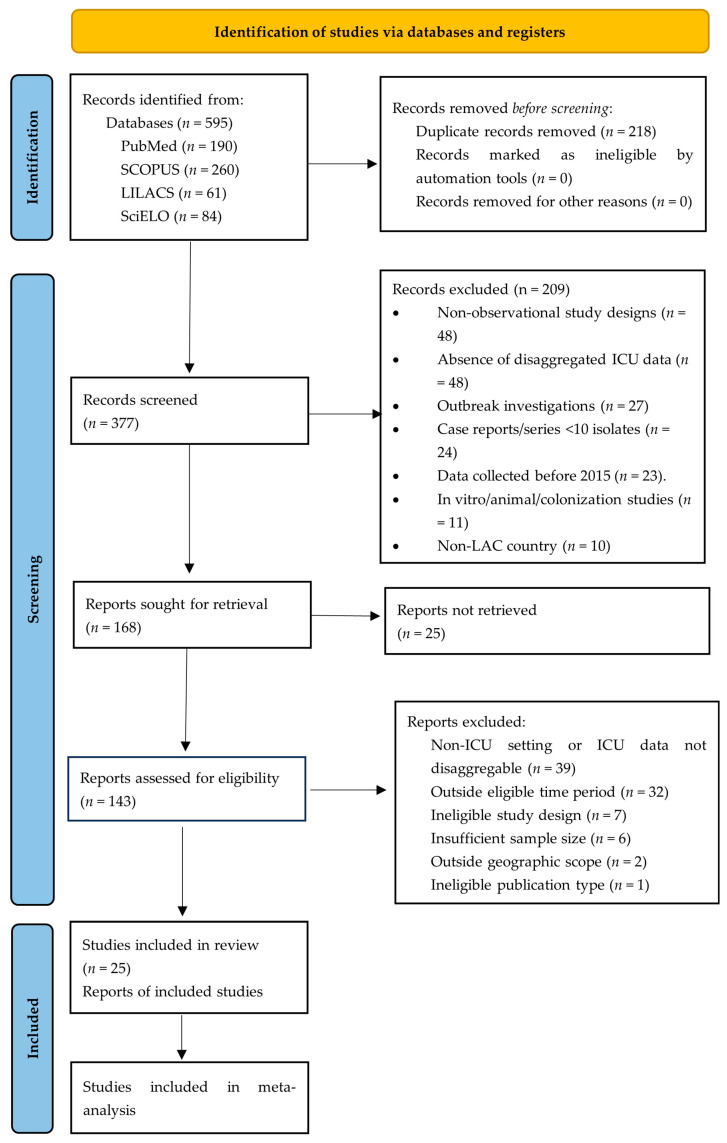
PRISMA 2020 flow diagram of study identification and selection. Flow diagram summarizing the search, screening, and eligibility assessment for studies reporting CR-GNB from ICU in LAC.

The full-text evaluation resulted in the exclusion of 143 articles. Finally, this systematic review included 25 studies. Among these, 12 studies met the eligibility criteria for quantitative meta-analysis, providing 49 independent observations for prevalence estimation. The remaining 13 studies were excluded from meta-analysis but contributed to the narrative synthesis of molecular epidemiology. The study selection process is illustrated in Figure 1 along with the checklist (Supplementary Table S1) following PRISMA 2020 guidelines.

### 2.2. Characteristics of Included Studies

Table 1 summarizes the characteristics of all 25 studies included in the systematic review. Studies originated from eight countries representing three geographic subregions: South America contributed the largest proportion with studies from Brazil (*n* = 11) [15,23,27,28,29,30,31,32,33,34,35,36], Peru (*n* = 4) [31,37,38,39], Argentina (*n* = 2) [40,41], Ecuador (*n* = 2) [42,43], Colombia (*n* = 1) [44], and Paraguay (*n* = 1) [45]; North America was represented by Mexico (*n* = 3) [46,47,48]; and the Caribbean by the Dominican Republic (*n* = 1) [49].

Study designs included cross-sectional studies (*n* = 12), retrospective cohort studies (*n* = 4), prospective cohort studies (*n* = 2), and surveillance studies (*n* = 7). Most investigations were conducted in teaching or university hospitals (*n* = 17), with the remainder distributed among public hospitals (*n* = 4), private hospitals (*n* = 2), and mixed settings involving multiple institution types (*n* = 2). Single-center studies predominated (*n* = 18), while seven studies employed multicenter designs involving between seven and 43 participating institutions.

ICU types varied across studies: adult ICUs (*n* = 10), mixed adult and pediatric ICUs (*n* = 9), neonatal ICUs (*n* = 3), and pediatric ICUs (*n* = 3). Sample sources included multiple specimen types in most studies (*n* = 17), followed by blood cultures exclusively (*n* = 3), and clinical isolates from various sites (*n* = 3). Antimicrobial susceptibility testing was performed using automated systems in most studies (*n* = 20), with disk diffusion (*n* = 3) and broth microdilution (*n* = 2) employed less frequently. Clinical breakpoints were interpreted according to the Clinical and Laboratory Standards Institute (CLSI) guidelines (*n* = 19), the European Committee on Antimicrobial Susceptibility Testing (EUCAST) (*n* = 3), and the Brazilian Committee on Antimicrobial Susceptibility Testing (BrCAST) (*n* = 3).

Among the 12 studies included in meta-analysis, study periods spanned from 2015 to 2024. These studies collectively reported 5092 total isolates, of which 1911 were carbapenem-resistant. These studies provided species-stratified data with explicit denominators permitting calculation of carbapenem resistance prevalence. The 13 studies contributing solely to narrative synthesis were excluded from quantitative pooling due to absence of total isolate denominators (*n* = 8), study designs limited to selected carbapenem-resistant isolates precluding prevalence estimation (*n* = 4), or colonization-based sampling strategies (*n* = 1).

The target bacterial species reported included *K. pneumoniae* (11 studies), *A. baumannii* (8 studies), *P. aeruginosa* (7 studies), *E. coli* (7 studies), and *E. cloacae* complex (6 studies).

### 2.3. Risk of Bias Assessment

Methodological quality was assessed using the Joanna Briggs Institute (JBI) Critical Appraisal Checklist for Studies Reporting Prevalence Data [50]. Overall quality was high, with JBI scores ranging from 62.50% to 100% (median: 93.75%). Eleven studies (91.7%) were classified as high-quality (≥70% criteria met), while one study [32] demonstrated moderate quality (62.50%) due to limitations in the sampling frame representativeness and outcome measurement validity. Three studies [23,30,40] achieved perfect scores of 100%. Common methodological limitations across studies included unclear or inconsistent application of statistical analysis methods and response rate reporting. Detailed risk of bias assessments are provided in Table S2.

### 2.4. Inter-Rater Agreement

Inter-rater agreement for study selection was evaluated using Cohen’s kappa coefficient. Based on the two-category classification system, the observed agreement was 80.1% with an expected agreement of 54.6%, yielding a Cohen’s kappa of 0.562. This value indicates moderate agreement according to Landis and Koch interpretation criteria. Of 377 records screened, 286 resulted in concordant decisions between reviewers, while 91 discrepancies required resolution. A total of 75 true discrepancies were adjudicated by the third reviewer (Table S3).

### 2.5. Overall Prevalence of CR-GNB

**Figure 2:**
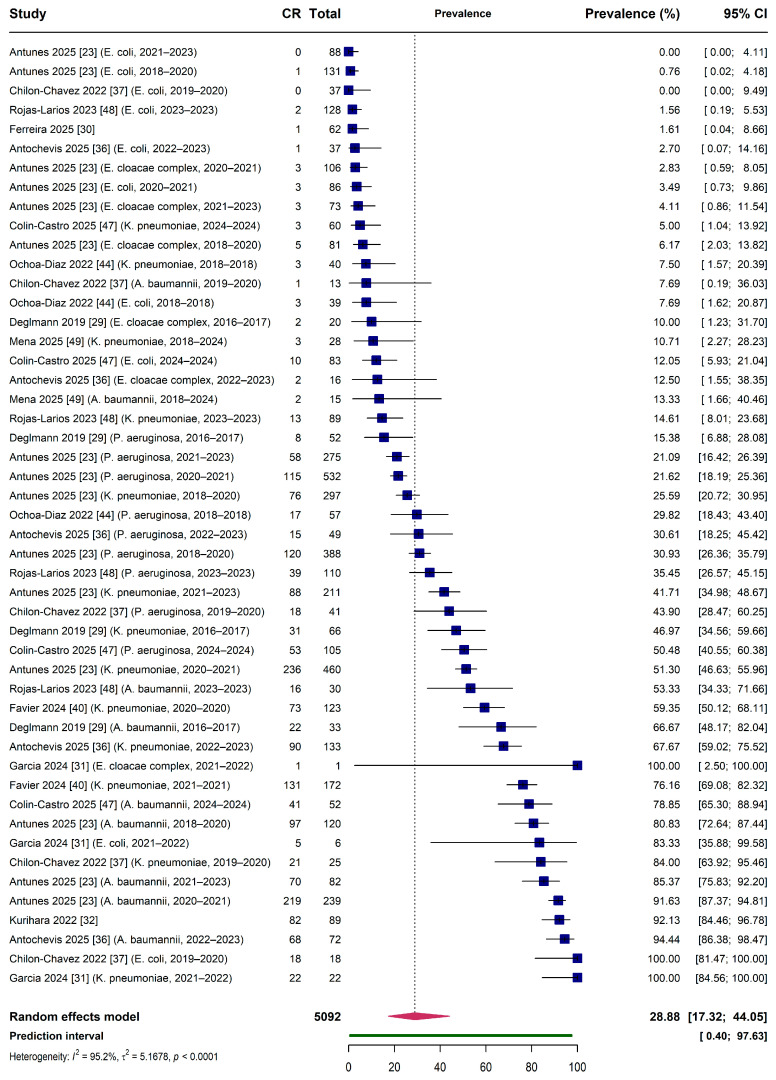
Forest plot of pooled carbapenem resistance prevalence among Gram-negative bacilli in Latin American and Caribbean intensive care units. Random-effects meta-analysis of 52 study-bacteria combinations from 12 studies (*n* = 5092 isolates) demonstrating pooled CR prevalence across all species. Individual study estimates are displayed with 95% C.I.; the pooled prevalence was 28.88% (95% CI: 17.32–44.05%) with substantial heterogeneity (I^2^ = 95.2%, τ^2^ = 5.17, *p* < 0.0001) and a wide P.I. (0.40–97.63%).

The pooled prevalence of carbapenem resistance among Gram-negative bacilli in Latin American and Caribbean ICUs was 28.88% (95% CI: 17.32–44.05%) based on 49 observations from 12 studies (Figure 2). The analysis employed a three-level generalized linear mixed model with random effects at both the observation and study levels to account for hierarchical data structure and multiple observations per study. Substantial heterogeneity was observed across studies (I^2^ = 95.24%), with between-study variance (τ^2^ = 5.1678) indicating considerable variability in true prevalence across settings. The 95% prediction interval ranged from 0.40% to 97.63%, reflecting the wide range of true prevalence values expected in future studies conducted under similar conditions.

The substantial heterogeneity observed (I^2^ = 95.24%, τ^2^ = 2.27) likely reflects genuine epidemiological variation across settings rather than a solely methodological artifact. Clinical contributors to this heterogeneity include differential ICU admission criteria across institutions, with tertiary referral centers typically managing higher-acuity patients with greater prior healthcare exposure. Variable implementation of antimicrobial stewardship programs—ranging from comprehensive multidisciplinary teams to minimal or absent programs—creates differential selection pressure for resistant organisms. Heterogeneous infection prevention practices, including variable adherence to contact precautions and active surveillance cultures, further contribute to institutional differences in CR-GNB colonization pressure.

Methodological sources of heterogeneity encompass differences in susceptibility testing approaches. While 83% of included studies utilized automated systems (VITEK, MicroScan, Phoenix), variability exists in instrument calibration, inoculum standardization, and incubation conditions. The application of different clinical breakpoints—with CLSI predominating (79%) but EUCAST and regional standards (BrCAST) also employed—affects resistance classification, particularly for isolates near breakpoint thresholds. Inconsistent molecular confirmation of carbapenemase production means that some studies may include isolates with non-carbapenemase resistance mechanisms (porin loss, efflux overexpression), while others restricted analysis to molecularly confirmed carbapenemase producers.

The prediction interval of 0.40–97.63% provides clinically meaningful context for this heterogeneity, indicating that a future study conducted in any LAC ICU could plausibly observe CR-GNB prevalence anywhere within this wide range depending on local epidemiology and institutional factors.

### 2.6. Prevalence by Bacterial Species

**Figure 3:**
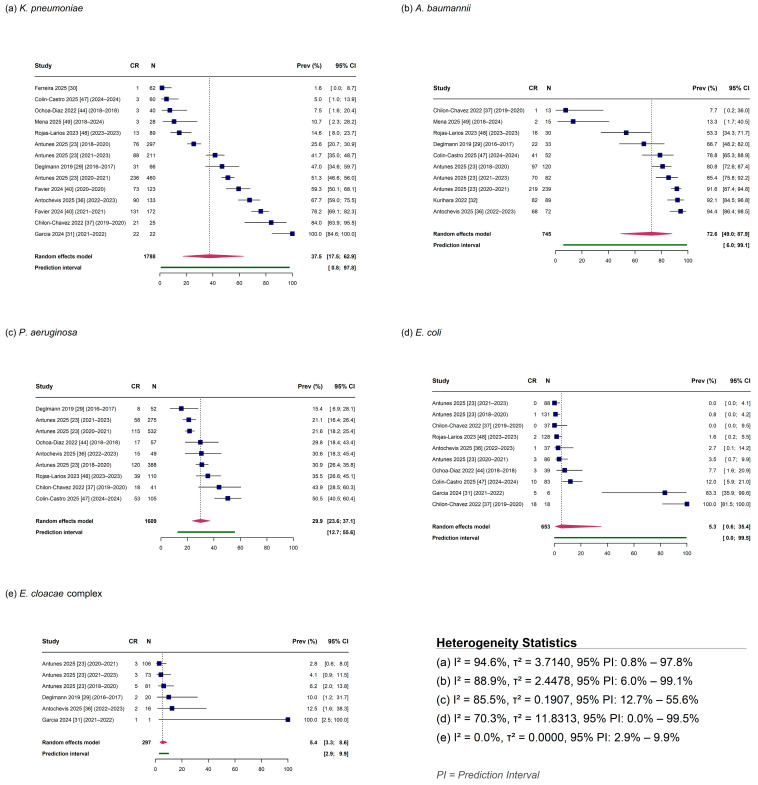
Species-stratified forest plots of carbapenem resistance prevalence among CR-GNB in LAC ICU. Subgroup meta-analyses stratified by bacterial species: (**a**) *K. pneumoniae* (pooled prevalence 37.5%, 95% CI: 17.5–62.9%, I^2^ = 94.6%); (**b**) *A. baumannii* (72.6%, 95% CI: 49.0–87.9%, I^2^ = 88.9%); (**c**) *P. aeruginosa* (29.9%, 95% CI: 22.6–37.1%, I^2^ = 85.5%); (**d**) *E. coli* (5.3%, 95% CI: 0.6–35.4%, I^2^ = 70.3%); and (**e**) *E. cloacae* complex (5.4%, 95% CI: 3.3–8.6%, I^2^ = 0.0%). Heterogeneity statistics and P.I. are provided for each species.

Species-specific analyses revealed marked differences in carbapenem resistance prevalence across the five target pathogens (Table 2; Figure 3).

*Acinetobacter baumannii* exhibited the highest pooled CR prevalence at 72.58% (95% CI: 49.04–87.92%) based on 10 observations from eight studies (Figure 3b). Substantial heterogeneity was observed (I^2^ = 88.94%; τ^2^ = 2.4478), and the 95% prediction interval extended from 5.99% to 99.10%, indicating considerable uncertainty in expected prevalence for future studies.

*Klebsiella pneumoniae* demonstrated intermediate prevalence at 37.48% (95% CI: 17.47–62.92%) across 14 observations from 11 studies (Figure 3a). This species showed the highest heterogeneity among those analyzed (I^2^ = 94.64%; τ^2^ = 3.714) and the widest prediction interval (0.79–97.83%), suggesting substantial between-study variability in carbapenem resistance rates.

*Pseudomonas aeruginosa* showed a pooled prevalence of 29.93% (95% CI: 23.64–37.09%) based on nine observations from seven studies (Figure 3c). While substantial heterogeneity remained (I^2^ = 85.51%; τ^2^ = 0.1907), this species exhibited a narrower prediction interval (12.72–55.61%) compared to other organisms, suggesting somewhat greater consistency in prevalence estimates across settings.

*Escherichia coli* and *Enterobacter cloacae* complex demonstrated markedly lower carbapenem resistance prevalence. *E. coli* showed a pooled prevalence of 5.26% (95% CI: 0.56–35.43%) across 10 observations from seven studies (Figure 3d), with moderate heterogeneity (I^2^ = 70.29%; τ^2^ = 11.8313) and an extremely wide prediction interval encompassing near-zero to near-complete resistance (0–99.52%). *E. cloacae* complex exhibited the lowest prevalence at 5.39% (95% CI: 3.33–8.61%) based on six observations from four studies (Figure 3e). Notably, this species showed no detectable heterogeneity (I^2^ = 0%; τ^2^ = 0), yielding the narrowest prediction interval among all species analyzed (2.86–9.93%).

### 2.7. Prevalence by COVID-19 Pandemic Period

**Figure 4:**
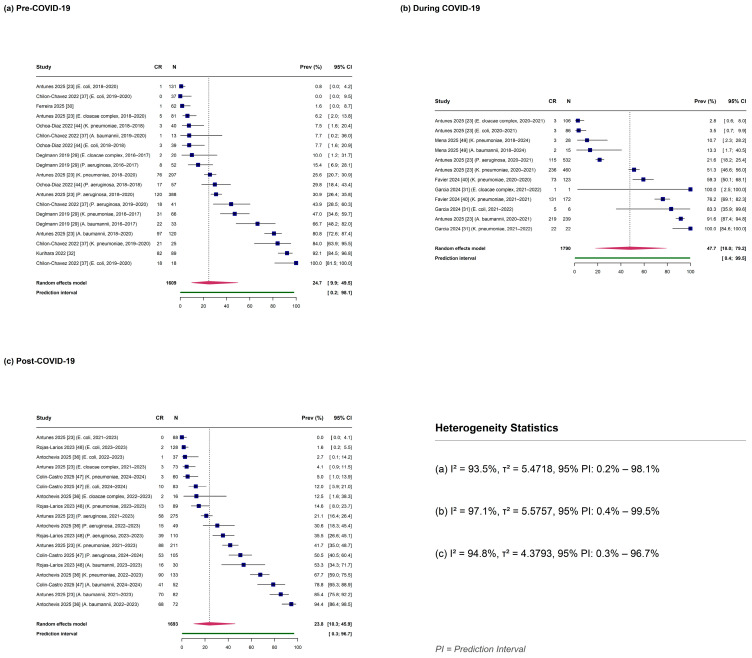
Temporal trends in CR prevalence stratified by COVID-19 pandemic period. Forest plots depicting pooled carbapenem resistance prevalence across three temporal periods: (**a**) pre-COVID-19 (<2020): 24.7% (95% CI: 9.9–49.5%, I^2^ = 93.5%); (**b**) during COVID-19 (2020–2021): 47.7% (95% CI: 18.0–79.2%, I^2^ = 97.1%); and (**c**) post-COVID-19 (≥2022): 23.8% (95% CI: 10.3–45.9%, I^2^ = 94.8%).

CR prevalence was examined across three pandemic periods defined by study midpoint (Table 3; Figure 4 and Figure S1). The highest pooled prevalence was observed during the COVID-19 pandemic period (study midpoint 2020–2021.5), at 47.72% (95% CI: 17.97–79.18%; *n* = 12 observations from four studies), with high heterogeneity (I^2^ = 97.13%) and a wide prediction interval of 0.40–99.53%.

Pre-COVID-19 studies (midpoint before 2020) demonstrated a pooled prevalence of 24.68% (95% CI: 9.88–49.47%) across 19 observations from six studies, with I^2^ = 93.48% and prediction interval of 0.21–98.09%. Post-COVID-19 studies (midpoint 2022 or later) showed similar prevalence to the pre-pandemic period at 23.79% (95% CI: 10.31–45.87%) based on 18 observations from four studies (I^2^ = 94.75%; prediction interval: 0.33–96.71%).

The during-COVID-19 period estimate (47.72%, 95% CI: 17.97–79.18%) should be interpreted with caution given the limited number of contributing studies (*n* = 4) and observations (*n* = 12). The wide confidence interval reflects substantial uncertainty inherent to this limited sample.

### 2.8. Stratified Analyses

#### 2.8.1. COVID-19 Period by Bacterial Species

Stratified analysis by COVID-19 period and bacterial species was performed for strata with three or more observations (Table 4). *K. pneumoniae* showed notable temporal variation: pre-COVID-19 prevalence was 23.08% (95% CI: 4.96–63.32%; *n* = 5; I^2^ = 92.11%), increasing substantially to 65.96% (95% CI: 24.60–92.01%; *n* = 5; I^2^ = 91.29%) during COVID-19, and returning to 26.13% (95% CI: 8.19–58.38%; *n* = 4; I^2^ = 96.00%) post-COVID-19.

*A. baumannii* prevalence was 65.92% (95% CI: 25.42–91.65%; *n* = 4; I^2^ = 87.92%) pre-COVID-19 and 81.99% (95% CI: 64.17–92.04%; *n* = 4; I^2^ = 85.79%) post-COVID-19; data during the COVID-19 period were insufficient for pooled analysis. *P. aeruginosa* showed relatively stable prevalence across periods: 29.45% (95% CI: 21.82–38.45%; *n* = 4; I^2^ = 65.39%) pre-COVID-19 and 33.40% (95% CI: 23.29–45.30%; *n* = 4; I^2^ = 90.30%) post-COVID-19, with insufficient data during COVID-19.

*E. coli* prevalence was 8.10% (95% CI: 0.03–96.87%; *n* = 4; I^2^ = 27.66%) pre-COVID-19 and 2.14% (95% CI: 0.43–9.90%; *n* = 4; I^2^ = 65.76%) post-COVID-19, with insufficient data during COVID-19. *E. cloacae* complex had insufficient observations across all COVID-19 periods for stratified analysis.

#### 2.8.2. Bacterial Species by Geographic Region

Stratified analysis by bacterial species and geographic region was limited to South America due to insufficient observations in North America and the Caribbean (Table S4). Within South America, *A. baumannii* showed the highest regional prevalence at 80.69% (95% CI: 57.75–92.74%; *n* = 7 observations from five studies; I^2^ = 85.60%; PI: 8.58–99.47%). *K. pneumoniae* prevalence in South America was 49.65% (95% CI: 23.87–75.63%; *n* = 11 observations from eight studies; I^2^ = 94.32%; PI: 1.21–98.75%).

*P. aeruginosa* showed lower prevalence at 26.22% (95% CI: 21.33–31.76%; *n* = 7 observations from five studies; I^2^ = 74.82%; PI: 14.12–43.43%). *E. coli* prevalence was 5.25% (95% CI: 0.24–56.05%; *n* = 8 observations from five studies; I^2^ = 68.32%; PI: 0–99.95%), and *E. cloacae* complex demonstrated the lowest prevalence at 5.39% (95% CI: 3.33–8.61%; *n* = 6 observations from four studies; I^2^ = 0%; PI: 2.86–9.93%).

### 2.9. Subgroup ICU Type

Analysis by ICU type showed comparable prevalence between adult ICUs (29.50%; 95% CI: 15.00–49.81%; *n* = 27 observations; I^2^ = 96.27%) and mixed adult–pediatric ICUs (34.36%; 95% CI: 14.72–61.36%; *n* = 19 observations; I^2^ = 93.47%). Neonatal ICUs demonstrated substantially lower prevalence at 5.96% (95% CI: 2.09–15.83%; *n* = 3 observations; I^2^ = 44.02%), representing the only subgroup with heterogeneity below 50%.

### 2.10. Sensitivity Analyses

Sensitivity analyses were performed to assess the robustness of the pooled estimate (Table S5). The main analysis prevalence of 28.88% remained stable across multiple analytical approaches. Excluding observations with 0% and 100% prevalence (*n* = 5 excluded) yielded 27.89% (95% CI: 17.72–40.99%; I^2^ = 95.74%).

Restricting to observations with sample sizes of at least 20 isolates (*n* = 6 excluded) resulted in 26.63% (95% CI: 15.50–41.80%; I^2^ = 95.77%), while restricting to at least 30 isolates (*n* = 10 excluded) yielded 24.37% (95% CI: 13.96–39.01%; I^2^ = 96.07%). Excluding observations with fewer than 5 isolates (*n* = 1 excluded) produced 28.06% (95% CI: 16.73–43.10%; I^2^ = 95.34%). All sensitivity analyses yielded estimates within 5 percentage points of the main analysis, indicating robust results.

### 2.11. Influence Diagnostics and Leave-One-Out Analysis

Leave-one-out analysis assessed the influence of individual studies on the pooled estimate (Table S6; Figure S2). Prevalence estimates ranged from 25.14% (excluding Garcia et al. 2024 [31]) to 34.55% (excluding Antunes et al. 2025 [23]). The largest influence was observed for Antunes et al. 2025 [23], which contributed 15 observations; its exclusion increased the pooled prevalence by 5.67 percentage points. Garcia 2024 [31] showed the opposite effect, with its exclusion decreasing prevalence by 3.74 percentage points. All other studies changed the pooled estimate by 1.6 percentage points or less when excluded.

Formal influence diagnostics were calculated for all 49 observations using Cook’s distance and studentized residuals (Table S7). Using distribution-based thresholds, no observations exceeded both thresholds simultaneously, indicating no highly influential outliers. Individual observations with elevated studentized residuals greater than 1.5 included several from Antunes et al. 2025 [23] (*A. baumannii* across multiple periods) and observations from Antochevis et al. 2025 [36] (*A. baumannii*), García et al. 2024 [31] (*K. pneumoniae*), and Chilon-Chavez et al. 2022 [37] (*E. coli*), though none met criteria for classification as highly influential.

### 2.12. Publication Bias Assessment

Publication bias was assessed using Doi plots and the Luis Furuya-Kanamori (LFK) index (Figures S3–S8). Major asymmetry (|LFK| > 2) was detected for *K. pneumoniae* (LFK = 6.877) and *A. baumannii* (LFK = 4.925). Minor asymmetry (1 < |LFK| ≤ 2) was observed for *P. aeruginosa* (LFK = −1.69) and *E. coli* (LFK = 1.741). *E. cloacae* complex showed no asymmetry (LFK = 0.887).

Peters’ test was performed for species with at least 10 studies. For *A. baumannii*, Peters’ test was statistically significant (*p* = 0.0003), suggesting potential publication bias. *K. pneumoniae* (*p* = 0.7537) and *E. coli* (*p* = 0.0692) showed no significant asymmetry by Peters’ test. Peters’ test was not performed for *P. aeruginosa* (*n* = 9 observations) and *E. cloacae* complex (*n* = 6 observations) due to insufficient sample size per Cochrane guidelines requiring a minimum of 10 studies.

LFK index values indicated major asymmetry across all COVID-19 periods: pre-COVID-19 (LFK = 5.149), during COVID-19 (LFK = −2.21), and post-COVID-19 (LFK = 9.685). These findings should be interpreted cautiously given the inherent limitations of small-study effect assessments in prevalence meta-analyses with substantial heterogeneity.

### 2.13. Molecular Epidemiology of Carbapenemase Genes

Seventeen of the 25 included studies reported molecular characterization of carbapenemase genes, providing detailed epidemiological data synthesized by bacterial species (Table S8).

#### 2.13.1. *K. pneumoniae*

KPC-type carbapenemases, predominantly *bla*KPC-2, were identified as the most common carbapenemase in *K. pneumoniae* across multiple countries. In Brazil, do Valle Barroso et al. 2023 [27] detected *bla*KPC-2 in all 20 carbapenem-resistant pediatric ICU isolates, while Borelli et al. 2021 [28] characterized *bla*KPC-2 on IncX3 plasmids with high structural conservation suggesting horizontal gene transfer potential. Lorenzoni et al. 2017 [34] reported 84% *bla*KPC prevalence in adult ICU isolates from southern Brazil.

In Peru, García et al. 2024 [31] and García-Cedrón et al. 2023 [38] documented 100% KPC detection among carbapenem-resistant *K. pneumoniae* isolates. In Ecuador, Mejía-Limones et al. 2024 [42] reported the first identification of *bla*KPC-3 in the country within a pediatric ICU setting, alongside predominant *bla*KPC-2 (35.7% of analyzed isolates). Soria-Segarra et al. 2020 [43] documented complete KPC carriage in colonized patients across seven Ecuadorian ICUs, with ST258 as the predominant clone and evidence of intra- and inter-hospital clonal transmission documented by PFGE.

Metallo-β-lactamases were documented in Argentina by Favier 2024 et al. [40], who reported emergence of MBL-producing strains including NDM following ceftazidime-avibactam introduction. Co-carriage of KPC with MBL was observed in 12 isolates, and *OXA-163* was detected in three isolates. Vargas et al. 2022 [41] characterized diverse clonal lineages (ST17, ST13, ST2256, ST353, ST86) harboring *bla*KPC-2, frequently co-harboring ESBL genes (*bla*SHV-2, *bla*CTX-M-15), and identified OXA-48-like enzymes in three isolates.

#### 2.13.2. *A. baumannii*

OXA-23-like carbapenemases predominated across the region. Chagas et al. 2025 [15] documented *bla*OXA-23 in 15/16 carbapenem-resistant *A. baumannii* isolates from Brazilian ICUs. Sánchez-Urtaza et al. 2025 [45] reported 100% *bla*OXA-23 carriage in Paraguayan isolates, identifying two international clones: IC2 (ST2) carrying *bla*OXA-23 in Tn2006 and IC5 (ST79) with *bla*OXA-23 in Tn2008. Lima et al. 2020 [33] detected OXA-23-like genes in 72% of Brazilian ICU isolates.

Castillo-Bejarano et al. 2023 [46] documented the first report of *bla*OXA-24 and *bla*IMP co-carriage in a pediatric population in Latin America and the Caribbean. All 21 isolates harbored *bla*OXA-24, with 12/21 (57%) additionally carrying *bla*IMP, predominantly identified during August–October 2020. Vargas et al. 2022 [41] in Argentina detected *bla*IMP-1 in 6/9 isolates, OXA-48-like in 2/9, and *bla*VIM-1 in one isolate.

#### 2.13.3. *P. aeruginosa*

Carbapenemase prevalence in *P. aeruginosa* was notably lower than in other species. García-Cedrón et al. 2023 [38] reported NDM as the predominant carbapenemase in Peru (11/16 isolates), with OXA-48 (4/16) and KPC (1/16) detected at lower frequencies. Souza et al. 2021 [35] in Brazil identified *bla*KPC-2 in only 11% (3/28) of carbapenem-resistant isolates, while testing negative for VIM, IMP, NDM, OXA-48, SPM, and GIM. This finding suggests that OprD porin loss, rather than carbapenemase production, represents the primary carbapenem resistance mechanism in Brazilian *P. aeruginosa*. Lima et al. 2020 [33] similarly detected low rates of *bla*KPC-2 (2/11 isolates). Vargas et al. 2022 [41] identified *bla*VIM-2 in a single Argentine isolate.

#### 2.13.4. *E. coli*

*E. coli* generally exhibited lower carbapenemase prevalence. García-Cedrón et al. 2023 [38] in Peru detected NDM (5/7) and OXA-48 (2/7) in carbapenem-resistant isolates. Garcia et al. 2024 [31] documented NDM (4/6) and OXA-48-like (2/6) during the COVID-19 pandemic. Borelli et al. 2021 [28] characterized *bla*KPC-2 in a Brazilian isolate with 100% gene identity to *K. pneumoniae* strains from the same setting, suggesting inter-species horizontal gene transfer. Vargas et al. 2022 [41] documented OXA-48-like in two Argentine isolates, with one patient demonstrating triple colonization by *E. coli*, *K. pneumoniae*, and *A. baumannii* all carrying OXA-48-like enzymes.

#### 2.13.5. *E. cloacae* Complex

Limited molecular data were available for *E. cloacae* complex. Soria-Segarra et al. 2020 [43] documented 100% KPC carriage in eight colonized isolates from Ecuadorian ICUs. García-Cedrón et al. 2023 [38] and Garcia et al. 2024 [31] each detected NDM in single isolates from Peru. The scarcity of molecular data reflects the relatively low carbapenem resistance prevalence observed in this species.

#### 2.13.6. Notable Findings and Emerging Patterns

Multi-species transmission of carbapenemase genes within healthcare settings was documented in several studies. Vargas et al. 2022 [41] provided compelling evidence of cross-species dissemination, identifying OXA-48-like enzymes simultaneously in *K. pneumoniae*, *A. baumannii*, and *E. coli* from the same patient, suggesting horizontal gene transfer among gut colonizers. Borelli et al. 2021 [28] demonstrated 100% amino acid identity of *bla*OXA-1 genes across *E. coli*, *K. pneumoniae*, and *Morganella morganii* in a Brazilian ICU.

Geographic patterns revealed KPC predominance throughout South America with emerging NDM detection, particularly in Argentina following novel therapeutic introductions. Novel findings included the first report of *bla*KPC-3 in Ecuador [42] and the first documentation of *bla*OXA-24 with *bla*IMP co-carriage in a pediatric Latin American population [46].

#### 2.13.7. Synthesis of Molecular Epidemiological Patterns

Integration of molecular data across included studies reveals distinct species-specific resistance mechanisms and geographic dissemination patterns within LAC. For *K. pneumoniae*, KPC-type carbapenemases—predominantly *bla*KPC-2—represent the dominant resistance mechanism throughout South America, achieving endemic status in Brazil, Argentina, Colombia, and Peru, where KPC prevalence among carbapenem-resistant isolates exceeds 80% in studies with molecular characterization. The recent emergence of *bla*KPC-3 in Ecuador and increasing *bla*NDM detection in Argentina signal an evolving molecular landscape with important therapeutic implications, particularly regarding novel β-lactam/β-lactamase inhibitor combinations.

*A. baumannii* resistance across LAC is predominantly mediated by OXA-type carbapenemases, with *bla*OXA-23 identified in over 70% of molecularly characterized isolates. International clones IC2 (corresponding to MLST ST2) and IC5 (ST79) serve as the primary vehicles for regional dissemination, with documented intra- and inter-hospital transmission supporting the critical need for enhanced infection prevention measures. The near-universal presence of these clones across geographically dispersed institutions suggests successful regional establishment rather than repeated independent introductions.

*P. aeruginosa* presents a distinct resistance profile wherein non-carbapenemase mechanisms—particularly OprD porin loss and MexAB-OprM efflux pump overexpression—contribute substantially to carbapenem resistance. Carbapenemase detection rates in *P. aeruginosa* (11–35% where specifically tested) are notably lower than in Enterobacterales, suggesting different selective pressures and distinct resistance evolution pathways for this intrinsically resistant species.

A critical emerging phenomenon requiring urgent attention is carbapenemase co-production, documented in Argentina where KPC-NDM co-producing *K. pneumoniae* are increasingly identified following the introduction of ceftazidime-avibactam. This co-production severely restricts therapeutic options, as ceftazidime-avibactam and other novel β-lactam/β-lactamase inhibitor combinations effective against KPC-producers lack activity against metallo-β-lactamases. Evidence of inter-species horizontal gene transfer within ICU settings—demonstrated through identical carbapenemase gene sequences (including flanking mobile genetic elements) across *K. pneumoniae*, *E. coli*, and *A. baumannii* recovered from single patients—underscores the genetic fluidity of resistance determinants and the critical importance of comprehensive, species-agnostic infection control approaches.

## 3. Discussion

This systematic review and meta-analysis provides the first comprehensive, region-specific estimate of CR-GNB prevalence in ICU across LAC. The pooled prevalence of 28.88% (95% CI: 17.32–44.05%) positions LAC among the most severely affected global regions, substantially exceeding rates documented in Western Europe (2.3%) and North America (1.4–5.3%) [7,51]. The exceptionally high I^2^ value (95.24%) and expansive prediction interval (0.40–97.63%) underscore the profound heterogeneity characterizing resistance patterns across the region, reflecting diverse healthcare infrastructures, variable antimicrobial stewardship implementation, and differential selective pressures among participating centers.

Species-specific analyses revealed a distinctive hierarchy of carbapenem resistance. *A. baumannii* emerged as the predominant threat with a pooled prevalence of 72.58% (95% CI: 49.04–87.92%), followed by *K. pneumoniae* at 37.48% (95% CI: 17.47–62.92%) and *P. aeruginosa* at 29.93% (95% CI: 23.64–37.09%). Notably, *E. coli* (5.26%) and *E. cloacae* complex (5.39%) exhibited considerably lower resistance rates, suggesting differential ecological niches and transmission dynamics within ICU environments [13,15]. This species-specific gradient carries direct implications for empirical antimicrobial selection in critically ill patients throughout the region.

The CR-GNB prevalence documented in LAC ICUs demonstrates epidemiological patterns that diverge substantially from other global regions while converging with certain high-burden areas. Western European surveillance from 2019 to 2020 reported carbapenem-resistant Enterobacterales (CRE) prevalence of 2.3% in bloodstream infections and 0.6% in complicated urinary tract infections—markedly lower than our LAC estimates [51]. In contrast, African neonatal sepsis data revealed CR-GNB prevalence of 30.34% (95% CI: 22.03–38.64%), remarkably similar to our overall findings, suggesting that resource-limited healthcare settings share common resistance amplification mechanisms regardless of geographic location [52].

The geographic gradient within LAC merits particular attention. South America exhibited the highest pooled prevalence (31.81%), followed by North America—represented by Mexico—(22.65%), and the Caribbean (11.63%). This south–north gradient aligns with established surveillance data demonstrating differential endemicity patterns, wherein Brazil, Argentina, and Colombia function as epicenters harboring established high-risk clones and serving as reservoirs for regional dissemination [16,18]. Brazil alone accounts for approximately 73% of reported carbapenemase isolates in regional studies, reflecting both population density and healthcare system characteristics [53].

Comparison with other Southern Hemisphere regions reveals both similarities and distinctions in CR-GNB epidemiology. African surveillance data, though fragmented, indicate comparable resistance burdens in critical care settings. A recent meta-analysis of neonatal sepsis in sub-Saharan Africa reported CR-GNB prevalence of 30.34% (95% CI: 22.03–38.64%) [52], remarkably similar to our LAC estimate of 28.88%, suggesting that resource-limited healthcare settings share common resistance amplification mechanisms regardless of geographic location. Species-specific patterns also show parallels, with *A. baumannii* exhibiting the highest resistance rates in both regions (45.9% in African neonates versus 81.99% in general population in LAC ICUs) [52]. In contrast, Australian ICU surveillance demonstrates substantially lower carbapenem resistance rates, with CR-Enterobacterales prevalence typically below 0.5% and *P. aeruginosa* resistance rates of approximately 3.1% [54]. This disparity likely reflects differences in antimicrobial stewardship implementation, infection prevention infrastructure, and healthcare system resources. A distinctive feature of Australian epidemiology is the predominance of the blaIMP-4 gene, representing over 50% of carbapenemases in CPE, a locally evolved carbapenemase endemic at low levels along the eastern coast [54]. It is also arguable that geographic isolation of the Australian continent may play a significant role to the observed resistant patterns. The convergence of LAC and African resistance patterns, contrasted with substantially lower Australian rates, underscores the critical role of healthcare infrastructure investment and coordinated infection control strategies in containing CR-GNB dissemination.

The *A. baumannii* prevalence of 72.58% warrants particular emphasis as a species-specific epidemiological outlier. Regional surveillance consistently reports carbapenem non-susceptibility rates between 79.3% and 89.2% for this pathogen in LAC ICU settings, whereas Asia-Pacific data range from 17% to 50% depending on the subregion [7,26]. This near-total resistance effectively removes carbapenems as a viable therapeutic option for empiric therapy in ventilator-associated pneumonia or catheter-associated bloodstream infections caused by Acinetobacter, forcing reliance on potentially toxic alternatives including polymyxins and tigecycline.

The exceptionally high CRAB prevalence (72.58%) carries profound clinical and infection control implications for LAC ICUs. CRAB infections represent the fourth-leading cause of death attributable to antimicrobial resistance globally [55]. This near-total carbapenem resistance effectively eliminates this antibiotic class from empirical treatment algorithms for suspected *Acinetobacter* infections, including ventilator-associated pneumonia—one of the most common ICU-acquired infections where *A. baumannii* is a leading pathogen [56].

Clinicians must increasingly rely on alternative agents with significant limitations. The ESCMID and IDSA guidelines recommend combination therapy with at least two in vitro active antibiotics for severe CRAB infections [57,58]. Polymyxins (colistin, polymyxin B) carry substantial nephrotoxicity risk and achieve suboptimal pulmonary concentrations due to pharmacokinetic limitations [59]. High-dose tigecycline or minocycline may be considered as combination partners, though tigecycline monotherapy has been associated with higher mortality in pneumonia [58]. High-dose ampicillin-sulbactam exploits sulbactam’s intrinsic anti-*Acinetobacter* activity, though variable in vitro susceptibility limits its utility [57].

Sulbactam-durlobactam, approved by the FDA in May 2023, represents a significant therapeutic advance. In the ATTACK trial, sulbactam-durlobactam combined with imipenem-cilastatin demonstrated a 28-day mortality rate of 19% compared to 32% for colistin-based regimens in patients with CRAB pneumonia or bloodstream infections [60]. The 2024 IDSA guidance document now recommends sulbactam-durlobactam in combination with a carbapenem as the preferred first-line regimen for CRAB infections [58]. However, these novel agents face substantial access barriers in LAC settings due to high cost, delayed regulatory approval processes, and limited inclusion in national essential medicines lists [61].

From an infection prevention perspective, *A. baumannii* can survive for extended periods (≥20 days to several months) on dry hospital surfaces, including bed rails, medical equipment, and healthcare worker clothing [62]. Environmental contamination has been documented as an important reservoir in ICU outbreaks, necessitating strict contact precautions, dedicated equipment, and enhanced environmental cleaning protocols [63]. These data support prioritizing infection prevention investment, which may yield more immediate impact than antimicrobial stewardship alone for this intrinsically resistant pathogen.

The substantial statistical heterogeneity (I^2^ = 95.24%, τ^2^ = 5.1678) observed in this meta-analysis reflects genuine epidemiological variation rather than methodological artifact. Contemporary guidance on prevalence meta-analyses emphasizes that elevated I^2^ values in such contexts typically represent true between-study differences arising from diverse populations, settings, and temporal factors rather than indicating analytical invalidity [64,65]. The prediction interval of 0.40–97.63% provides a more clinically meaningful interpretation, indicating that a future study conducted in a LAC ICU could plausibly observe CR-GNB prevalence anywhere within this range.

Multiple factors contribute to this heterogeneity. ICU type demonstrated significant influence, with mixed adult-pediatric units exhibiting the highest prevalence (34.36%), followed by adult ICUs (29.50%) and neonatal units (5.96%). These age-related differences are corroborated by recent Argentine multicenter surveillance data: the PREV-AR-P study documented MDRO infection prevalence of 4.6% in 50 pediatric ICUs, substantially lower than the 15.1% observed in adult ICUs within the same surveillance network [10,66]. Study design similarly affected estimates, with prospective surveillance studies yielding higher prevalence (38.35%) compared to retrospective analyses (25.17%). These methodological differences likely reflect ascertainment bias, wherein prospective designs capture a more complete picture of colonization and infection burden [19].

Healthcare infrastructure heterogeneity across LAC represents another substantial contributor. Delayed diagnostic capabilities, variable isolation room availability, differential antimicrobial stewardship implementation, and inconsistent infection prevention practices collectively create a fragmented resistance landscape. Countries with robust surveillance networks—including Colombia’s Instituto Nacional de Salud and Argentina’s ANLIS-Malbrán—demonstrate capacity for comprehensive resistance monitoring, whereas resource-limited settings may experience underreporting or incomplete characterization [19].

The temporal stratification revealed a pronounced pandemic effect on CR-GNB prevalence. Studies conducted during the COVID-19 pandemic documented prevalence of 47.72%, compared to 24.68% in pre-pandemic periods and 23.79% in post-pandemic assessments. This pattern aligns with global observations of antimicrobial resistance amplification during the pandemic, driven by multiple converging factors [25,67]. While our temporal analysis suggests pandemic-associated resistance amplification consistent with global reports, the limited sample size during the COVID-19 period (four studies, 12 observations) and the exceptionally wide confidence interval (17.97–79.18%) preclude definitive conclusions. This finding should be considered hypothesis-generating rather than confirmatory. Future studies specifically designed to capture pandemic-era resistance dynamics in LAC ICUs are needed to establish whether the observed elevation represents a true temporal trend or reflects sampling variation.

Empirical antibiotic prescribing surged during pandemic early phases, with documented increases in carbapenem, ceftriaxone, and azithromycin consumption across 13 LAC countries [68]. A direct statistical correlation was established between consumption patterns and resistance emergence: each unit increase in defined daily dose of beta-lactams and fluoroquinolones associated with 11–22% increases in resistant *E. coli* and *K. pneumoniae* bloodstream infections [69]. Concurrently, infection prevention resources were diverted toward aerosol transmission prevention, often compromising contact precautions essential for multidrug-resistant organism containment.

Italian COVID-19 ICU data documented carbapenem-resistant *A. baumannii* (CRAB) colonization in 65% of mechanically ventilated patients, with whole-genome sequencing confirming single-strain predominance indicative of nosocomial amplification [70]. Brazilian data demonstrated a 4.7-fold increase in CRAB incidence density during the pandemic (*p* < 0.001), with rates remaining elevated in the immediate post-pandemic period [71]. This “post-pandemic plateau” suggests that resistance gains achieved during healthcare system disruption may represent a new endemic baseline rather than a transient phenomenon.

The clinical burden associated with CR-GNB infections in LAC ICUs is substantial. Mortality rates range from 27% to 64% across regional studies, with carbapenem-resistant Enterobacterales bloodstream infections demonstrating particularly elevated lethality [10,72]. A multicenter LAC study reported 28-day mortality of 64% for carbapenemase-producing Enterobacterales bacteremia compared to 30% for non-carbapenemase-producing infections, underscoring the therapeutic failure associated with resistance [73]. Pediatric populations exhibit similarly concerning outcomes, with pooled mortality of 34% in CRE bloodstream infections [9].

Economic analyses reveal direct costs 50.4% higher for resistant versus susceptible infections, driven by expensive second-line antimicrobials, prolonged ICU stays (excess 4–7.4 days), and increased diagnostic requirements [11,74].

These findings carry direct implications for empirical therapy algorithms. With overall CR-GNB prevalence approaching 30% in LAC ICUs—and *A. baumannii* resistance exceeding 70%—carbapenem monotherapy can no longer serve as reliable empirical coverage for suspected Gram-negative infections in critically ill patients. Risk stratification incorporating prior colonization status, recent healthcare exposure, and local resistance patterns becomes essential for antimicrobial selection [48].

Although molecular characterization fell outside the primary scope of this prevalence meta-analysis, synthesis of regional surveillance data provides essential context. KPC-type carbapenemases remain the dominant resistance mechanism in LAC Enterobacterales, with blaKPC-2 and blaKPC-3 variants exhibiting extraordinarily high prevalence: Argentina 76.7%, Brazil 79.2%, Colombia 53.3%, and Puerto Rico 85.3% [53,75]. However, NDMs demonstrate concerning emergence, particularly in Guatemala (74.3%), Mexico (44.1%), and Venezuela (51.4%), representing a molecular epidemiological shift with therapeutic implications [76].

For *A. baumannii*, OXA-type carbapenemases—particularly OXA-23 and OXA-40—dominate the resistance landscape, with international clone IC2 demonstrating widespread global distribution and IC5 exhibiting LAC-specific predominance [77,78]. *P. aeruginosa* resistance is primarily mediated by VIM and IMPs, with KPC emergence reported in specific geographic foci [79].

A critical emerging concern is carbapenemase co-production. Argentina’s post-COVID-19 RECAPT-AR study documented equivalent circulation of NDM and KPC for the first time, with high frequency of KPC-NDM co-producers predominantly in *K. pneumoniae* [19]. This co-production phenomenon severely restricts therapeutic options, as novel β-lactam/β-lactamase inhibitor combinations active against KPC (ceftazidime-avibactam, meropenem-vaborbactam) lack activity against metallo-β-lactamases. Aztreonam-avibactam demonstrated exceptional activity (96–98% susceptibility) against all carbapenemase classes in this context, representing a potential therapeutic solution pending regional availability [19].

This systematic review represents the first comprehensive, LAC-specific meta-analysis of CR-GNB prevalence in ICU settings. Methodological strengths include prospective registration (PROSPERO CRD420251177826), adherence to PRISMA 2020 guidelines, and application of JBI methodology for prevalence reviews. The statistical approach employed a generalized linear mixed model (GLMM) with logit transformation, recognized as the optimal method for proportional meta-analyses by avoiding boundary issues and providing appropriate handling of studies with extreme proportions [80,81].

The comprehensive search strategy encompassed four databases (PubMed, Scopus, LILACS, SciELO), including regional platforms essential for capturing LAC-specific literature potentially absent from global indices. Dual independent screening with substantial inter-rater agreement (κ = 0.562) and rigorous risk of bias assessment using the JBI checklist—with 11 of 12 studies (91.7%) classified as high quality—strengthen confidence in the included evidence. Sensitivity analyses demonstrated robust findings, with pooled estimates varying less than 5 percentage points across analytical permutations.

Several limitations warrant consideration when interpreting these findings. The substantial heterogeneity (I^2^ = 95.24%), while anticipated in prevalence meta-analyses, limits the precision of pooled estimates and necessitates cautious application to specific clinical settings. The prediction interval spanning 0.40–97.63% reflects genuine uncertainty regarding expected prevalence in any individual LAC ICU.

Geographic representation, though spanning eight countries, remains incomplete. Notable gaps include Central American nations (except Nicaragua), several Caribbean island states, and underrepresentation of smaller South American countries. Brazil and Colombia contributed disproportionately to the evidence base, potentially biasing estimates toward patterns characteristic of these larger healthcare systems. Publication bias was detected for *A. baumannii* analyses (Peters’ test *p* = 0.0003), suggesting potential overestimation of prevalence for this pathogen.

The assessment of small-study effects for *A. baumannii* revealed major asymmetry on Doi plot analysis (LFK index = 4.925), suggesting potential publication bias. Peters’ regression test was not performed for this species (k = 8 unique studies) following Cochrane Handbook recommendations that regression-based tests require at least 10 studies to achieve adequate statistical power [82]. The observed asymmetry may reflect selective publication of studies documenting exceptionally high resistance rates due to perceived novelty or may indicate that institutions experiencing severe CRAB problems are more motivated to publish surveillance data. Consequently, the pooled *A. baumannii* prevalence estimate (72.58%) should be interpreted with caution, as it may represent an overestimate of the true regional prevalence.

Methodological variability among primary studies introduces additional uncertainty. Susceptibility testing methods ranged from automated systems to disk diffusion, with variable breakpoint application. Resistance definitions were not uniformly standardized across studies, and molecular confirmation of carbapenemase production was inconsistently performed. The temporal span (2015–2025) encompasses evolving epidemiology, with older studies potentially underestimating current resistance burdens while pandemic-era studies may overestimate endemic prevalence.

In addition, a formal GRADE assessment was not conducted, as standardized guidance for applying this framework to prevalence systematic reviews remains unavailable. The methodological quality of included studies was instead evaluated using the JBI Critical Appraisal Checklist for Studies Reporting Prevalence Data, which is specifically designed for this review type.

Future research priorities should address the identified geographic gaps through multicenter surveillance studies in underrepresented countries. Standardization of susceptibility testing methodologies and resistance definitions would enhance comparability across regional studies. Prospective cohort studies evaluating clinical outcomes stratified by carbapenemase genotype would inform treatment algorithms, while economic evaluations of stewardship interventions would support resource allocation decisions in healthcare systems with competing priorities.

In conclusion, this systematic review documents CR-GNB prevalence of 28.88% in LAC ICUs, with *A. baumannii* exhibiting the highest species-specific resistance (72.58%). These findings position LAC among the most severely affected global regions and carry immediate implications for empirical antimicrobial therapy, infection prevention priorities, and antimicrobial stewardship implementation. The COVID-19 pandemic amplified pre-existing resistance patterns, establishing a potentially elevated endemic baseline. Coordinated regional action integrating enhanced surveillance, stewardship expansion, and equitable access to novel therapeutics remains essential to address this critical public health threat.

## 4. Materials and Methods

### 4.1. Protocol and Registration

This systematic review and meta-analysis was conducted in accordance with the Preferred Reporting Items for Systematic Reviews and Meta-Analyses (PRISMA) 2020 guidelines [83], the Joanna Briggs Institute (JBI) Manual for Evidence Synthesis with specific reference to the chapter on systematic reviews of prevalence and incidence [84], and relevant sections of the Cochrane Handbook for Systematic Reviews of Interventions [82]. The review protocol was prospectively registered in the International Prospective Register of Systematic Reviews (PROSPERO) under registration number CRD420251177826 prior to commencing the literature search. Amendments to the original protocol were made during the conduct of this review and are documented in PROSPERO (version 2.0). Key methodological changes included: (1) the use of Maximum Likelihood instead of DerSimonian-Laird estimation for between-study variance; (2) Doi plots with LFK index and Peters’ test instead of funnel plots with Egger’s test for publication bias assessment; and (3) non-application of the GRADE framework for certainty assessment, as GRADE was developed for intervention reviews and its application to prevalence studies remains methodologically controversial.

### 4.2. Eligibility Criteria

Study eligibility was determined using the Condition, Context, and Population (CoCoPop) framework as recommended by the JBI for prevalence reviews [84].

#### 4.2.1. Condition

The condition of interest was carbapenem resistance in Gram-negative bacilli (CR-GNB). Phenotypic resistance was defined according to Clinical and Laboratory Standards Institute (CLSI) or European Committee on Antimicrobial Susceptibility Testing (EUCAST) clinical breakpoints. Isolates were considered carbapenem-resistant if they demonstrated non-susceptibility (intermediate or resistant) to at least one carbapenem agent: imipenem, meropenem, or doripenem for all Gram-negative bacilli, with ertapenem additionally considered for *Enterobacterales*.

#### 4.2.2. Context

Eligible studies were conducted in ICUs, including adult, pediatric, mixed, or neonatal units, within hospital-based settings across LAC. Countries were classified according to the Pan American Health Organization (PAHO) geographic subregions: South America (Argentina, Bolivia, Brazil, Chile, Colombia, Ecuador, Guyana, Paraguay, Peru, Suriname, Uruguay, Venezuela), Central America (Belize, Costa Rica, El Salvador, Guatemala, Honduras, Nicaragua, Panama), North America (Mexico), and the Caribbean (Cuba, Dominican Republic, Haiti, Jamaica, Puerto Rico, Trinidad and Tobago, and other island nations). Studies exclusively investigating outbreak situations or environmental samples were excluded.

#### 4.2.3. Population

The population comprised clinical isolates of Gram-negative bacilli recovered from patients admitted to ICUs. Target organisms included *Klebsiella pneumoniae*, *Acinetobacter baumannii* (including the *A. baumannii-calcoaceticus* complex), *Pseudomonas aeruginosa*, *Escherichia coli*, and the *Enterobacter cloacae* complex. Studies were required to report both the numerator (number of carbapenem-resistant isolates) and denominator (total number of isolates tested) to enable prevalence calculation.

#### 4.2.4. Study Design and Publication Criteria

Observational studies employing cross-sectional, cohort, or surveillance designs were eligible for inclusion. The search was restricted to studies published between 1 January 2015 and 31 October 2025 to capture contemporary prevalence data relevant to current clinical practice and resistance epidemiology. No language restrictions were applied. Case reports, case series with fewer than 10 isolates, outbreak investigations, studies without disaggregated ICU data, in vitro studies, environmental sampling studies, and publications reporting duplicate patient populations were excluded.

The temporal restriction to January 2015 onwards was established based on three considerations: (1) the standardization of carbapenem susceptibility testing following updates to Clinical and Laboratory Standards Institute (CLSI) and European Committee on Antimicrobial Susceptibility Testing (EUCAST) breakpoints that occurred in the preceding years, ensuring methodological comparability across included studies; (2) the need to capture contemporary resistance epidemiology relevant to current clinical practice and infection control policy development; and (3) the inclusion of the COVID-19 pandemic period (2020–2024), which has been hypothesized to significantly impact antimicrobial resistance patterns in intensive care settings due to increased antibiotic utilization and disruption of stewardship programs.

### 4.3. Information Sources and Search Strategy

A comprehensive literature search was conducted across four electronic databases: PubMed/MEDLINE, Scopus, Literatura Latinoamericana y del Caribe en Ciencias de la Salud (LILACS), and Scientific Electronic Library Online (SciELO). The search was executed on 15 November 2025, following a two-week indexing lag to ensure complete capture of studies published through October 2025. Only formally published studies with complete bibliographic information were eligible; no ‘in press’ or ‘ahead of print’ publications were included. The search strategy combined Medical Subject Headings (MeSH) terms and free-text keywords encompassing three conceptual domains: (1) carbapenem resistance and related terminology (“carbapenem-resistant,” “carbapenemase”, “CRE,” “CRAB”, “CRPA”); (2) target organisms (*Klebsiella*, *Acinetobacter*, *Pseudomonas*, *Enterobacterales*, “Gram-negative”); and (3) clinical setting (“intensive care”, “ICU”, “critical care”). Geographic filters were applied to restrict results to LAC countries [85]. The complete search strategies for all databases, including database-specific syntax adaptations, are provided in Supplementary Table S9. Reference lists of included studies and relevant systematic reviews were manually screened to identify additional eligible publications.

### 4.4. Study Selection Process

Retrieved citations were imported into a Microsoft Excel spreadsheet (Microsoft Corporation, Redmond, WA, USA) for systematic screening. Following removal of duplicates, two independent reviewers (JDR, MCMV) performed title and abstract screening according to pre-defined eligibility criteria. Potentially relevant articles proceeded to full-text assessment, conducted independently by the same reviewers. Disagreements at any stage were resolved through discussion and consensus or, when necessary, adjudication by a third reviewer (JCBA). Inter-rater reliability was assessed using Cohen’s kappa coefficient, with values interpreted as: <0.20 (poor), 0.21–0.40 (fair), 0.41–0.60 (moderate), 0.61–0.80 (substantial), and >0.80 (almost perfect agreement) [27].

### 4.5. Data Extraction

Data were extracted using a standardized, pre-piloted extraction form by two independent reviewers (JDR, MCMV). Extracted variables included: study identifiers (first author, publication year, country, study period); setting characteristics (ICU type, hospital type, number of beds); population characteristics (patient age group, sample size); resistance data (phenotypic testing methods, interpretive guidelines used, number of carbapenem-resistant isolates, total isolates tested, stratified by bacterial species where available); and methodological quality indicators. For multi-center studies or those reporting data across multiple time periods, separate observations were extracted when disaggregated data permitted.

Study authors were contacted via electronic mail when clarification of reported data was required; non-respondents after two attempts were documented, and available data were extracted as reported.

### 4.6. Risk of Bias Assessment

Methodological quality was evaluated using the JBI Critical Appraisal Checklist for Studies Reporting Prevalence Data, comprising nine items assessing sample frame appropriateness, sampling methodology, sample size adequacy, description of subjects and setting, coverage of the identified sample, validity of outcome measurement methods, standardization of measurement, statistical analysis appropriateness, and response rate adequacy [50]. Two reviewers (JDR, MCMV) independently assessed each included study, with disagreements resolved by a third reviewer (JCBA). Each item was scored as “Yes” (criterion met), “No” (criterion not met), “Unclear” (insufficient information), or “Not applicable.” An overall quality score was calculated as the proportion of applicable items receiving a “Yes” response (range: 0–1). Studies were not excluded based on risk of bias scores; instead, the influence of methodological quality on pooled estimates was examined through sensitivity analyses and meta-regression. The distribution of risk of bias scores is presented in Supplementary Table S2.

### 4.7. Data Synthesis and Statistical Analysis

#### 4.7.1. Software and Packages

All statistical analyses were performed using R version 4.4.1 (R Foundation for Statistical Computing, Vienna, Austria) [86]. The primary meta-analysis was conducted using the *meta* package (version 8.2.1) with the metaprop() function [87]. Advanced meta-regression and multilevel modeling utilized the *metafor* package (version 4.8.0) [88]. Publication bias assessment employed the *metasens* package (version 1.5.3) for Doi plot generation and Luis Furuya-Kanamori (LFK) index calculation [89]. For data handling, we used *tidyverse* (version 2.0.0) [90], while for generating figures, we used *ggplot2* (version 3.5.2) [91].

#### 4.7.2. Meta-Analytic Model

We employed a two-level multilevel GLMM to account for the hierarchical data structure, where multiple observations (species-specific data points) were nested within studies [80,92]. This one-stage approach directly models the binomial distribution of event counts without requiring variance-stabilizing transformations or continuity corrections for studies with zero events [93]. The model structure comprised: Level 1—individual observations (bacteria-specific data points within studies); Level 2—studies (accounting for within-study correlation among multiple observations from the same study). Maximum likelihood (ML) estimation was implemented via metaprop() with method = “GLMM” and method.tau = “ML” parameters [80,94].

#### 4.7.3. Effect Measure and Back-Transformation

Prevalence estimates were calculated on the logit scale and subsequently back-transformed to the proportion scale using the inverse logit function (plogis). Results are reported as percentages with accompanying 95% CI. Prediction intervals (95% PI) were calculated and reported to convey the expected range of true effects in similar future studies, following recommendations by IntHout and colleagues [65].

#### 4.7.4. Heterogeneity Assessment

Statistical heterogeneity was assessed using multiple complementary approaches. Cochran’s Q statistic with associated *p*-value tested the null hypothesis of homogeneity, with statistical significance set at α = 0.10 given the test’s low power with few studies [95]. The I^2^ statistic quantified the proportion of total variability attributable to between-study heterogeneity, interpreted according to Cochrane thresholds: 0–40% (might not be important), 30–60% (moderate heterogeneity), 50–90% (substantial heterogeneity), and 75–100% (considerable heterogeneity) [82]. The between-study variance (τ^2^) was estimated using maximum likelihood. PI provided a complementary assessment of heterogeneity by illustrating the distribution of true effects across the study population.

#### 4.7.5. Subgroup Analyses

Pre-specified subgroup analyses were performed when at least two observations were available per stratum. Stratification variables included: bacterial species (*K. pneumoniae*, *A. baumannii*, *P. aeruginosa*, *E. coli*, *E. cloacae* complex); geographic region (South America, Central America, Mexico, Caribbean); ICU type (adult, mixed, neonatal); patient population (adult, mixed, neonatal); and COVID-19 pandemic period based on study midpoint date (pre-COVID-19: midpoint < 2020; during COVID-19: 2020 ≤ midpoint < 2022; post-COVID-19: midpoint ≥ 2022). Heterogeneity within and between subgroups was evaluated using the Q statistic. Geographic analysis was conducted at the regional level rather than by individual country due to sample size constraints.

#### 4.7.6. Sensitivity Analyses

Sensitivity analyses were performed to assess the robustness of pooled estimates: (1) excluding observations with extreme proportions (0% or 100% prevalence); (2) restricting to observations with N ≥ 20 isolates; (3) restricting to observations with N ≥ 30 isolates; and (4) excluding very small samples (N < 5). Results were considered robust if pooled prevalence estimates varied by fewer than 5 percentage points across sensitivity analyses.

#### 4.7.7. Leave-One-Out and Influence Diagnostics

Leave-one-out analysis was performed by iteratively excluding each study and recalculating the pooled estimate. Studies were flagged as influential if their exclusion altered the pooled prevalence by more than 2 percentage points. Additionally, formal influence diagnostics were calculated for the main analysis, including Cook’s distance (threshold > 0.5), standardized residuals (|value| > 2 warranting inspection), leverage (hat values), and DFFITS statistics [88]. Studies exceeding both Cook’s distance and studentized residual thresholds were classified as “highly influential”; those exceeding only one threshold were considered “potentially influential”.

#### 4.7.8. Exploratory Meta-Regression

Exploratory meta-regression analyses were conducted using multilevel random-effects models (rma.mv function in *metafor*) with observations nested within studies [88]. The following moderators were examined: study midpoint year (continuous) to assess temporal trends; risk of bias score (continuous) to examine the relationship between methodological quality and reported prevalence; and COVID-19 period (categorical) to test for discrete differences between pandemic periods. Effect sizes were calculated as logit-transformed proportions with corresponding sampling variances. ML estimation was used. Statistical significance was set at α = 0.05 for all analyses.

### 4.8. Publication Bias Assessment

Publication bias was assessed using Doi plots and the Luis Furuya-Kanamori (LFK) index, following recommendations by Furuya-Kanamori and colleagues for meta-analyses of proportions [96]. The LFK index was interpreted as: |LFK| ≤ 1.0 indicating no asymmetry; 1.0 < |LFK| ≤ 2.0 indicating minor asymmetry; and |LFK| > 2.0 indicating major asymmetry suggestive of publication bias. This approach was selected over traditional funnel plots and Egger’s test, which have demonstrated poor performance characteristics for binary outcomes and proportions [97]. Additionally, Peters’ test was performed when ≥10 studies were available [98,99].

Small-study effects and potential publication bias were assessed using Doi plots with the LFK index, where values exceeding ±1 indicate minor asymmetry and values exceeding ±2 indicate major asymmetry [82]. Peters’ regression test was performed only for species with ≥10 unique contributing studies, following Cochrane Handbook recommendations that regression-based asymmetry tests have insufficient power with fewer studies [82].

## 5. Conclusions

This systematic review and meta-analysis establishes that approximately 29% of Gram-negative bacilli isolated in LAC ICUs demonstrate carbapenem resistance, with *A. baumannii* representing the predominant resistance concern at nearly 73% prevalence. These findings position LAC among high-burden regions globally and underscore the urgent need for regional policies addressing antimicrobial stewardship, infection prevention, and surveillance strengthening. The substantial heterogeneity observed reflects modifiable factors related to healthcare infrastructure investment and intervention implementation. Concerted action by clinicians, infection preventionists, policymakers, and international partners is required to address this critical threat to patient safety in LAC critical care settings.

## Figures and Tables

**Table 1:** Characteristics of included studies reporting phenotypic CR-GNB from ICUs in LAC (2015–2025).

Author	Date	Country	Study Period	Study Design	Number of Centers	Sample Size	ICU Type	AST Method
Antunes et al. [23]	2025	Brazil	2018–2023	Retrospective cohort	8	3169	ICU	NR
do Valle Barroso et al. [27]	2023	Brazil	2016–2021	Retrospective cross-sectional	1	NR	Mixed	Automated, DD, MIC
Borelli et al. [28]	2021	Brazil	2018–2018	Cross-sectional	1	NR	ICU	Automated
Castillo Bejarano et al. [46]	2023	Mexico	2017–2022	Surveillance	1	NR	Mixed	Automated
Chagas et al. [15]	2025	Brazil	2019–2023	Cross-sectional	1	NR	ICU	Automated
Chilon-Chavez et al. [37]	2022	Peru	2019–2020	Cross-sectional	1	134	Mixed	Automated
Colín-Castro et al. [47]	2025	Mexico	2024–2024	Surveillance	41	300	Mixed	Automated
Deglmann et al. [29]	2019	Brazil	2016–2017	Cross-sectional	1	171	ICU	NR
Favier et al. [40]	2024	Argentina	2020–2021	Surveillance	1	295	ICU	Automated
Ferreira et al. [30]	2025	Brazil	2015–2022	Retrospective cohort	1	62	NICU	Automated
García et al. [31]	2024	Peru	2021–2022	Cross-sectional	1	29	ICU	Automated
García-Cedrón et al. [38]	2023	Peru	2021–2022	Cross-sectional	1	NR	ICU	Automated
Kurihara et al. [32]	2022	Brazil	2015–2017	Retrospective surveillance	1	89	Mixed	Automated and MIC
Lima et al. [33]	2020	Brazil	2015–2017	Prospective cohort	1	NR	ICU	Automated, DD, E-test
Lorenzoni et al. [34]	2017	Brazil	2015	Cross-sectional	1	NR	ICU	Automated
Mejía-Limones et al. [42]	2024	Ecuador	2020–2021	Cross-sectional	1	NR	PICU	Automated
Mena et al. [49]	2025	Dominican Republic	2018–2024	Retrospective surveillance	1	43	NICU	Automated
Ochoa-Diaz et al. [44]	2022	Colombia	2018	Cross-sectional	21	136	ICU	Automated
Pérez-Lazo et al. [39]	2021	Peru	2015–2018	Retrospective cohort	1	NR	ICU	Automated
Rojas-Larios et al. [48]	2023	Mexico	2023–2023	Surveillance	43	357	Mixed	Automated and DD
Sánchez-Urtaza et al. [45]	2025	Paraguay	2023–2024	Cross-sectional	1	NR	Mixed	Automated
Soria-Segarra et al. [43]	2020	Ecuador	2016–2016	Prospective cohort	7	NR	ICU	DD
Souza et al. [35]	2021	Brazil	2015–2016	Cross-sectional	1	NR	ICU	MIC
Vargas et al. [41]	2022	Argentina	2018–2019	Surveillance	1	NR	ICU	Automated
Antochevis et al. [36]	2025	Brazil	2022–2023	Prospective cohort	14	307	Mixed	Automated, DD

ICU: Intensive care units; PICU: Pediatric Intensive Care Unit; NICU: Neonatal Intensive Care Unit; NR: not reported; MIC: Minimum Inhibitory Concentration; DD: Disk Diffusion.

**Table 2:** Species-specific pooled prevalence of carbapenem-resistant Gram-negative bacilli in ICUs across Latin America and the Caribbean, with heterogeneity and small-study effects metrics.

Bacteria	*n* (%)	Obs.	Studies	Prevalence	C.I.	P.I.	I^2^ (%)	τ^2^	LFK Index	Asymmetry	Peters’ *p*
*K. pneumoniae*	1788 (35.11)	14	11	37.48%	17.47–62.92	0.79–97.83	94.64	3.714	6.877	Major asymmetry	0.7537
*A. baumannii*	745 (14.63)	10	8	72.58%	49.04–87.92	5.99–99.10	88.94	2.4478	4.925	Major asymmetry	NA
*P. aeruginosa*	1609 (31.60)	9	7	29.93%	23.64–37.09	12.72–55.61	85.51	0.1907	−1.69	Minor asymmetry	NA
*E. coli*	653 (12.82)	10	7	5.26%	0.56–35.43	0.00–99.52	70.29	11.8313	1.741	Minor asymmetry	NA
*E. cloacae* complex	297 (5.83)	6	4	5.39%	3.33–8.61	2.86–9.93	0.00	0.00	0.887	No asymmetry	NA

C.I.: Confidence interval; P.I.: Prediction interval; NA = Peters’ test not performed.

**Table 3:** Temporal trends in CR-GNB prevalence stratified by COVID-19 pandemic periods in LAC in ICUs.

Period	Obs.	Studies	Frame Time	Prevalence	C.I.	P.I.	I^2^	τ^2^	LFK Index	Asymmetry
Pre-COVID-19	19	6	2016–2019.5	24.68%	9.88–49.47	0.21–98.09	93.48%	5.4718	5.149	Major asymmetry
During COVID-19	12	4	2020–2021.5	47.72%	17.97–79.18	0.4–99.53	97.13%	5.5757	−2.21	Major asymmetry
Post-COVID-19	18	4	2022–2024	23.79%	10.31–45.87	0.33–96.71	94.75%	4.3793	9.685	Major asymmetry

C.I.: Confidence interval; P.I.: Prediction interval.

**Table 4:** Species-specific prevalence of CR-GNB stratified by COVID-19 pandemic period in LAC in ICUs.

Bacteria	Period	Prevalence	C.I.	P.I.	I^2^	Obs.	Studies
*A. baumannii*	Pre-COVID-19	65.92	25.42–91.65	0.45–99.88	87.92%	4	4
*A. baumannii*	Post-COVID-19	81.99	64.17–92.04	16.11–99.08	85.79%	4	4
*E. coli*	Pre-COVID-19	8.10	0.03–96.87	0.00–100	27.66%	4	3
*E. coli*	Post-COVID-19	2.14	0.43–9.9	0.02–73.12	65.76%	4	4
*K. pneumoniae*	Pre-COVID-19	23.08	4.96–63.32	0.08–99.08	92.11%	5	5
*K. pneumoniae*	During COVID-19	65.96	24.6–92.01	0.53–99.86	91.29%	5	4
*K. pneumoniae*	Post-COVID-19	26.13	8.19–58.38	0.27–97.9	96%	4	4
*P. aeruginosa*	Pre-COVID-19	29.45	21.82–38.45	11.21–57.99	65.39%	4	4
*P. aeruginosa*	Post-COVID-19	33.40	23.29–45.3	8.49–73.06	90.3%	4	4

C.I.: Confidence interval; P.I.: Prediction interval.

## Data Availability

The complete dataset, including extracted data, and RoB assessments, is available in the Supplementary Materials.

## Supplementary Materials

The following supporting information can be downloaded at: https://www.mdpi.com/article/10.3390/antibiotics15020209/s1. Table S1. PRISMA 2020 checklist for systematic reviews; Table S2. Risk of bias assessment using JBI Critical Appraisal Checklist for Studies Reporting Prevalence Data for each included study; Table S3. Inter-rater agreement for title/abstract screening, including Cohen’s kappa coefficients; Table S4. Prevalence of carbapenem resistance stratified by bacterial species and geographic region (South America); Table S5. Sensitivity analysis results for the pooled prevalence estimate under different exclusion criteria; Table S6. Leave-one-out analysis showing the influence of each study on the pooled prevalence estimate; Table S7. Influence diagnostics including Cook’s distance, standardized residuals, leverage, and DFFIT values for each study–bacteria observation; Table S8. Characteristics of studies included for narrative synthesis of carbapenem resistance genes, including gene families, variants, detection methods, and co-carriage patterns; Table S9. Complete search strategies for PubMed, Scopus, and LILACS/SciELO databases; Figure S1. Forest plot of pooled carbapenem resistance prevalence with 95% confidence and prediction intervals stratified by COVID-19 pandemic period; Figure S2. Leave-one-out sensitivity analysis for the overall pooled carbapenem resistance prevalence estimate; Figure S3. Doi plot for publication bias assessment in the overall meta-analysis (LFK index = 10.90); Figure S4. Doi plot for publication bias assessment in *Escherichia coli* subgroup analysis (LFK index = 1.74); Figure S5. Doi plot for publication bias assessment in *Klebsiella pneumoniae* subgroup analysis (LFK index = 6.88); Figure S6. Doi plot for publication bias assessment in *Acinetobacter baumannii* subgroup analysis (LFK index = 4.93); Figure S7. Doi plot for publication bias assessment in Enterobacter cloacae complex subgroup analysis (LFK index = 0.89); Figure S8. Doi plot for publication bias assessment in Pseudomonas aeruginosa subgroup analysis (LFK index = −1.89).
