# Supplementary material for: Prevalence of Carbapenem-Resistant Gram-Negative Bacilli from Intensive Care Units from Latin America and the Caribbean: A Systematic Review and Meta-Analysis
Source: Antibiotics (Basel). 2026 Feb 14;15(2):209. doi: 10.3390/antibiotics15020209 (PMC12937657; doi:10.3390/antibiotics15020209)
Supplement: Supplementary file 1 [file antibiotics-15-00209-s001.zip › antibiotics-4092130-supplementary.pdf]

Supplementary tables

Table S1. PRISMA 2020 checklist.

Section and Topic	Item #	Checklist item	Location where item is reported
**TITLE**			
Title	1	Identify the report as a systematic review.	Title, Page 1, Lines 2-4
**ABSTRACT**			
Abstract	2	See the PRISMA 2020 for Abstracts checklist.	Lines 21-28
**INTRODUCTION**			
Rationale	3	Describe the rationale for the review in the context of existing knowledge.	Lines 113-119
Objectives	4	Provide an explicit statement of the objective(s) or question(s) the review addresses.	Lines 120-125
**METHODS**			
Eligibility criteria	5	Specify the inclusion and exclusion criteria for the review and how studies were grouped for the syntheses.	Lines 719-764
Information sources	6	Specify all databases, registers, websites, organisations, reference lists and other sources searched or consulted to identify studies.	Lines 765-780
Search strategy	7	Present the full search strategies for all databases, registers and websites, including any filters and limits used.	Table S9

Selection process	8	Specify the methods used to decide whether a study met the inclusion criteria of the review.	Lines 782-790
Data collection process	9	Specify the methods used to collect data from reports, including how many reviewers collected data from each report.	Lines 782-790
Data items	10a	List and define all outcomes for which data were sought.	Lines 791-803
Data items	10b	List and define all other variables for which data were sought.	Lines 791-803
Study risk of bias assessment	11	Specify the methods used to assess risk of bias in the included studies.	Lines 804-817; Table S2
Effect measures	12	Specify for each outcome the effect measure(s) used in the synthesis or presentation of results.	Lines 839-844
Synthesis methods	13a	Describe the processes used to decide which studies were eligible for each synthesis.	Lines 791-800
Synthesis methods	13b	Describe any methods required to prepare the data for presentation or synthesis.	Lines 791-817
Synthesis methods	13c	Describe any methods used to tabulate or visually display results of individual studies and syntheses.	Lines 820-827; Figures 2-4
Synthesis methods	13d	Describe any methods used to synthesize results and provide a rationale for the choice(s).	Lines 828-837
Synthesis methods	13e	Describe any methods used to explore possible causes of heterogeneity among study results.	Lines 844-853

Synthesis methods	13f	Describe any sensitivity analyses conducted to assess robustness of the synthesized results.	Lines 864-869
Reporting bias assessment	14	Describe any methods used to assess risk of bias due to missing results in a synthesis.	Lines 888-902
Certainty assessment	15	Describe any methods used to assess certainty (or confidence) in the body of evidence for an outcome.	N/A - See response to reviewer; JBI tool used for individual studies (Table S2)
**RESULTS**			
Study selection	16a	Describe the results of the search and selection process, from the number of records identified to the number of studies included.	Lines 127-139; Figure 1
Study selection	16b	Cite studies that might appear to meet the inclusion criteria, but which were excluded, and explain why they were excluded.	Figure 1; Table S8.
Study characteristics	17	Cite each included study and present its characteristics.	Table 1; Table S8
Risk of bias in studies	18	Present assessments of risk of bias for each included study.	Lines 174-183; Table S2
Results of individual studies	19	For all outcomes, present, for each study: summary statistics and effect estimates with precision.	Lines 192-225; Tables 2-4; Figures 2-4
Results of syntheses	20a	For each synthesis, briefly summarise the characteristics and risk of bias among contributing studies.	Lines 174-183; Table S2
Results of syntheses	20b	Present results of all statistical syntheses conducted.	Lines 192-328; Tables 2-4

Results of syntheses	20c	Present results of all investigations of possible causes of heterogeneity among study results.	Lines 254-282; Table S3
Results of syntheses	20d	Present results of all sensitivity analyses conducted to assess the robustness of the synthesized results.	Lines 302-312; Tables S5-S6
Reporting biases	21	Present assessments of risk of bias due to missing results for each synthesis assessed.	Lines 329-344; Figures S3-S4
Certainty of evidence	22	Present assessments of certainty in the body of evidence for each outcome assessed.	N/A
**DISCUSSION**			
Discussion	23a	Provide a general interpretation of the results in the context of other evidence.	Lines 453-651
Discussion	23b	Discuss any limitations of the evidence included in the review.	Lines 652-692
Discussion	23c	Discuss any limitations of the review processes used.	Lines 652-692
Discussion	23d	Discuss implications of the results for practice, policy, and future research.	Lines 693-701
**OTHER INFORMATION**			
Registration and protocol	24a	Provide registration information for the review, including register name and registration number.	Lines 125; PROSPERO CRD420251177826
Registration and protocol	24b	Indicate where the review protocol can be accessed.	PROSPERO database

Registration and protocol	24c	Describe and explain any amendments to information provided at registration or in the protocol.	Lines 711-718
Support	25	Describe sources of financial or non-financial support for the review.	Author contributions section
Competing interests	26	Declare any competing interests of review authors.	Conflicts of Interest section
Availability of data, code and other materials	27	Report which of the following are publicly available and where they can be found: template data collection forms; data extracted from included studies; data used for all analyses; analytic code; any other materials used in the review.	Supplementary materials; Data available on request

N/A = Not applicable.

Table S2. Risk of bias assessment for selected studies.

Author-Year	Sample size	Q1	Q2	Q3	Q4	Q5	Q6	Q7	Q8	Q9	Score	Yes %	Study quality
Antunes et. al, 2025	9780	Yes	Yes	Yes	Yes	Yes	Yes	Yes	NA	Yes	100.00	100.00	High quality
Chilon-Chavez et. al, 2022	208	Yes	Yes	Yes	Yes	Yes	Unclear	Yes	NA	Yes	93.75	87.50	High quality
Colín-Castro et. al, 2025	300	Yes	Yes	Yes	Yes	Yes	Yes	Yes	Unclear	Yes	94.44	88.89	High quality
Deglmann et. al, 2019	171	Yes	Yes	Yes	Yes	Yes	Unclear	Yes	NA	Unclear	87.50	75.00	High quality
Favier et. al, 2024	295	Yes	Yes	Yes	Yes	Yes	Yes	Yes	NA	Yes	100.00	100.00	High quality
Ferreira et. al, 2025	62	Yes	Yes	Yes	Yes	Yes	Yes	Yes	NA	Yes	100.00	100.00	High quality
García et. al, 2024	41	Yes	Yes	Unclear	Yes	Yes	Yes	Yes	NA	Yes	93.75	87.50	High quality
Kurihara et. al, 2022	89	Yes	No	Yes	Yes	No	Unclear	Yes	NA	Yes	68.75	62.50	Moderate quality
Mena et. al, 2025	28	Yes	Yes	Yes	Yes	Yes	Unclear	Yes	NA	Yes	93.75	87.50	High quality
Ochoa-Diaz et. al, 2022	199	Yes	Yes	Yes	Yes	Yes	Unclear	Yes	NA	Yes	93.75	87.50	High quality
Rojas-Larios et. al, 2023	357	Yes	Yes	Yes	Yes	Unclear	Yes	Yes	Unclear	Yes	88.89	77.78	High quality
Zavascki et. al, 2025	495	Yes	Yes	Yes	Unclear	Yes	Yes	Yes	Unclear	Yes	88.89	77.78	High quality

Table S3. Inter-Rater Agreement for Title/Abstract Screening.

Metric	Value
Total records screened	377
Total agreements	286
Total discrepancies	91
Observed agreement (3-category)	75.9%
Expected agreement (3-category)	51.4%
Cohen's Kappa (3-category)	0.503
Interpretation (3-category)	Moderate agreement

Observed agreement (2-category)	80.1%
Expected agreement (2-category)	54.6%
Cohen's Kappa (2-category)	0.562
Interpretation (2-category)	Moderate agreement

Passed to full-text (agreement)	94
Excluded (agreement)	208
True discrepancies (3rd reviewer)	75

Kappa interpretation (Landis & Koch, 1977): <0.20 slight, 0.21-0.40 fair, 0.41-0.60 moderate, 0.61-0.80 substantial, >0.80 almost perfect. 2-category system treats Include-Doubt and Doubt-Include as agreements.

Table S4. Prevalence by bacteria by region.

Bacteria	Region	Prevalence	C.I.	P.I.	I2	Obs.	Studies
A. baumannii	South America	80.69%	57.75 - 92.74	8.58 - 99.47	85.6%	7	5
E. cloacae complex	South America	5.39%	3.33 - 8.61	2.86 - 9.93	0.00%	6	4
E. coli	South America	5.25%	0.24 - 56.05	0.00 - 99.95	68.32%	8	5
K. pneumoniae	South America	49.65%	23.87 - 75.63	1.21 - 98.75	94.32%	11	8
P. aeruginosa	South America	26.22%	21.33 - 31.76	14.12 - 43.43	74.82%	7	5

Table S5. Sensitivity analysis.

Analysis	Excluded	Included	Prevalence	C.I.	P.I.	I2
Main pooled prevalence (reference)	0	49	28.88%	17.32 - 44.05	0.4 - 97.63	95.24%
Excluding 0% and 100% prevalence	5	44	27.89%	17.72 - 40.99	0.76 - 95.1	95.74%
Only observations with N >= 20	6	43	26.63%	15.5 - 41.8	0.39 - 97.11	95.77%
Only observations with N >= 30	10	39	24.37%	13.96 - 39.01	0.41 - 96.16	96.07%
Excluding observations with N < 5	1	48	28.06%	16.73 - 43.1	0.39 - 97.52	95.34%

Table S6. Leave-one-out analysis.

Author & date	Obs. Excluded	Prevalence	C.I.	P.I.	I^2	τ^2	Prevalence change	I^2 change
Antunes 2025	15	34.55	19.43 - 53.61	0.53 - 98.12	92.43	4.9412	5.67	-2.81
Garcia 2024	3	25.14	15.00 - 38.99	0.42 - 96.42	95.53	4.6319	-3.74	0.29
Ochoa-Diaz 2022	3	30.44	17.83 - 46.87	0.37 - 98.08	95.41	5.4518	1.56	0.17
Favier 2024	2	27.32	15.93 - 42.72	0.35 - 97.61	94.95	5.2999	-1.56	-0.29
Kurihara 2022	1	27.39	16.34 - 42.13	0.40 - 97.25	95.09	4.9859	-1.49	-0.15
Ferreira 2025	1	30.31	18.37 - 45.67	0.45 - 97.65	95.28	5.0208	1.43	0.04
Rojas-Larios 2023	4	30.23	17.59 - 46.79	0.37 - 98.04	95.4	5.4206	1.35	0.16
Mena 2025	2	29.96	17.70 - 45.98	0.38 - 97.95	95.39	5.3664	1.08	0.15
Chilon-Chavez 2022	5	27.80	16.65 - 42.60	0.48 - 96.82	95.66	4.584	-1.08	0.42
Zavascki 2025	5	28.04	16.22 - 43.95	0.37 - 97.59	95.31	5.1785	-0.84	0.07
Deglmann 2019	4	28.76	16.41 - 45.35	0.32 - 98.07	95.52	5.6257	-0.12	0.28
Colin-Castro 2025	4	28.87	16.63 - 45.24	0.34 - 97.94	95.31	5.4579	-0.01	0.07

Table S7. Influence diagnostics.

Study	Effect	S.E.	weight_pct	Cook's distance	Std. residuals	Leverage	DFFIT	τ^2
Antunes 2025 (A. baumannii, 2021-2023)	1.7636	0.3124	2.19	0.038	1.33	0.0219	0.1967	3.83
Antunes 2025 (A. baumannii, 2018-2020)	1.4392	0.2319	2.22	0.0302	1.165	0.0222	0.1744	3.8722
Antunes 2025 (A. baumannii, 2020-2021)	2.3933	0.2336	2.22	0.0584	1.684	0.0222	0.2474	3.7204
Chilon-Chavez 2022 (A. baumannii, 2019-2020)	-2.4849	1.0408	1.76	0.0096	-0.738	0.0176	-0.0976	3.9432
Colin-Castro 2025 (A. baumannii, 2024-2024)	1.3157	0.3396	2.18	0.0263	1.091	0.0218	0.1625	3.8903
Deglmann 2019 (A. baumannii, 2016-2017)	0.6931	0.3693	2.17	0.0137	0.769	0.0217	0.1162	3.9534
Kurihara 2022	2.4608	0.3938	2.16	0.0576	1.696	0.0216	0.2457	3.7214
Mena 2025 (A. baumannii, 2018-2024)	-1.8718	0.7596	1.96	0.0045	-0.486	0.0196	-0.0662	3.9789
Rojas-Larios 2023 (A. baumannii, 2023-2023)	0.1335	0.366	2.17	0.0058	0.487	0.0217	0.0755	3.991
Zavascki 2025 (A. baumannii, 2022-2023)	2.8332	0.5145	2.11	0.0666	1.873	0.0211	0.2658	3.6679
Deglmann 2019 (E. cloacae complex, 2016-2017)	-2.1972	0.7454	1.97	0.0081	-0.643	0.0197	-0.0892	3.9609
Zavascki 2025 (E. cloacae complex, 2022-2023)	-1.9459	0.7559	1.96	0.0052	-0.521	0.0196	-0.0714	3.9753
Antunes 2025 (E. cloacae complex, 2021-2023)	-3.1499	0.5896	2.07	0.0273	-1.134	0.0207	-0.1659	3.872
Antunes 2025 (E. cloacae complex, 2018-2020)	-2.7213	0.4617	2.13	0.019	-0.934	0.0213	-0.1375	3.9151
Antunes 2025 (E. cloacae complex, 2020-2021)	-3.5361	0.5857	2.07	0.038	-1.333	0.0207	-0.1968	3.8223
Garcia 2024 (E. cloacae complex, 2021-2022)	1.0986	1.633	1.34	0.0081	0.761	0.0134	0.0899	3.9373
Antunes 2025 (E. coli, 2020-2021)	-3.3202	0.5877	2.07	0.0318	-1.222	0.0207	-0.1794	3.8511
Antunes 2025 (E. coli, 2021-2023)	-5.1761	1.4182	1.48	0.0501	-1.82	0.0148	-0.2271	3.7398
Antunes 2025 (E. coli, 2018-2020)	-4.8675	1.0038	1.79	0.0643	-1.874	0.0179	-0.2593	3.6901
Chilon-Chavez 2022 (E. coli, 2019-2020)	3.6109	1.4332	1.47	0.0468	1.863	0.0147	0.2192	3.7441
Chilon-Chavez 2022 (E. coli, 2019-2020)	-4.3175	1.4236	1.48	0.0317	-1.447	0.0148	-0.1794	3.824
Colin-Castro 2025 (E. coli, 2024-2024)	-1.9879	0.3372	2.19	0.007	-0.571	0.0219	-0.0829	3.9767
Garcia 2024 (E. coli, 2021-2022)	1.6094	1.0954	1.72	0.0209	1.097	0.0172	0.1446	3.894
Ochoa-Diaz 2022 (E. coli, 2018-2018)	-2.4849	0.6009	2.06	0.0133	-0.799	0.0206	-0.1147	3.9393
Rojas-Larios 2023 (E. coli, 2023-2023)	-4.1431	0.7127	1.99	0.0537	-1.616	0.0199	-0.2359	3.7469

Zavascki 2025 (E. coli, 2022-2023)	-3.5835	1.0138	1.78	0.0286	-1.251	0.0178	-0.1701	3.8525
Antunes 2025 (K. pneumoniae, 2021-2023)	-0.3348	0.1396	2.24	0.0018	0.257	0.0224	0.0424	4.0119
Antunes 2025 (K. pneumoniae, 2018-2020)	-1.0674	0.133	2.24	2.00E-04	-0.112	0.0224	-0.0131	4.0164
Antunes 2025 (K. pneumoniae, 2020-2021)	0.0522	0.0933	2.24	0.0053	0.454	0.0224	0.0717	3.9971
Chilon-Chavez 2022 (K. pneumoniae, 2019-2020)	1.6582	0.5455	2.09	0.0318	1.241	0.0209	0.1795	3.8567
Colin-Castro 2025 (K. pneumoniae, 2024-2024)	-2.9444	0.5923	2.06	0.0224	-1.03	0.0206	-0.1499	3.8953
Deglmann 2019 (K. pneumoniae, 2016-2017)	-0.1214	0.2466	2.21	0.0034	0.363	0.0221	0.0579	4.0039
Favier 2024 (K. pneumoniae, 2020-2020)	0.3784	0.1836	2.23	0.0093	0.618	0.0223	0.0956	3.9773
Favier 2024 (K. pneumoniae, 2021-2021)	1.1616	0.179	2.23	0.0238	1.022	0.0223	0.1544	3.9054
Ferreira 2025	-4.1109	1.0082	1.78	0.0415	-1.503	0.0178	-0.2061	3.7929
Garcia 2024 (K. pneumoniae, 2021-2022)	3.8067	1.4298	1.48	0.0507	1.95	0.0148	0.2287	3.7226
Mena 2025 (K. pneumoniae, 2018-2024)	-2.1203	0.611	2.05	0.0078	-0.619	0.0205	-0.0875	3.9665
Ochoa-Diaz 2022 (K. pneumoniae, 2018-2018)	-2.5123	0.6003	2.06	0.0137	-0.813	0.0206	-0.1168	3.937
Rojas-Larios 2023 (K. pneumoniae, 2023-2023)	-1.7658	0.3001	2.2	0.0045	-0.461	0.022	-0.0661	3.9903
Zavascki 2025 (K. pneumoniae, 2022-2023)	0.7386	0.1854	2.23	0.0152	0.802	0.0223	0.1226	3.9487
Antunes 2025 (P. aeruginosa, 2021-2023)	-1.3195	0.1478	2.24	0.0011	-0.239	0.0224	-0.0325	4.0106
Antunes 2025 (P. aeruginosa, 2018-2020)	-0.8035	0.1098	2.24	1.00E-04	0.021	0.0224	0.007	4.0186
Antunes 2025 (P. aeruginosa, 2020-2021)	-1.2882	0.1053	2.24	9.00E-04	-0.223	0.0224	-0.0302	4.0118
Chilon-Chavez 2022 (P. aeruginosa, 2019-2020)	-0.2451	0.3147	2.19	0.0024	0.3	0.0219	0.0482	4.0074
Colin-Castro 2025 (P. aeruginosa, 2024-2024)	0.019	0.1952	2.23	0.0048	0.435	0.0223	0.0687	3.9982
Deglmann 2019 (P. aeruginosa, 2016-2017)	-1.7047	0.3844	2.17	0.0037	-0.427	0.0217	-0.0605	3.9926
Ochoa-Diaz 2022 (P. aeruginosa, 2018-2018)	-0.8557	0.2895	2.2	0	-0.005	0.022	0.003	4.0165
Rojas-Larios 2023 (P. aeruginosa, 2023-2023)	-0.5991	0.1993	2.23	5.00E-04	0.124	0.0223	0.0224	4.0166
Zavascki 2025 (P. aeruginosa, 2022-2023)	-0.8183	0.31	2.2	0	0.014	0.022	0.0058	4.0163

Table S8. Characteristics of the studies included for narrative synthesis.

Study_ID	Country	Species	Gene family	Gene variant	Prevalence_Gene	Detection method	Co-carriage	Comments
Favier2024	Argentina	*K. pneumoniae*	MBL	*bla*MBL	NR	Immunochromatography	MBL+ESBL: 31; MBL+KPC: 3	Denominator not explicit; includes NDM; detected by NG-test CARBA5
Favier2024	Argentina	*K. pneumoniae*	KPC	*bla*KPC	NR	Immunochromatography	KPC+MBL: 3	Denominator not explicit
Favier2024	Argentina	*K. pneumoniae*	OXA-48-like	*bla*OXA-163	NR	Immunochromatography	NR	Denominator not explicit
Favier2024	Argentina	*K. pneumoniae*	MBL	*bla*MBL	NR	Immunochromatography	MBL+ESBL: 53; MBL+KPC: 12	Denominator not explicit; includes NDM; detected by NG-test CARBA5
Favier2024	Argentina	*K. pneumoniae*	KPC	*bla*KPC	NR	Immunochromatography	KPC+MBL: 12	Denominator not explicit
Favier2024	Argentina	*K. pneumoniae*	OXA-48-like	*bla*OXA-163	NR	Immunochromatography	NR	Denominator not explicit
Vargas2022	Argentina	*K. pneumoniae*	KPC	*bla*KPC-2	NR	PCR	KPC-2 + SHV-2 + CTX-M-15 in majority of isolates	KPC-2 isolates all associated with ESBL (SHV-2 and/or CTX-M-15); clones ST17, ST86, ST2256, ST13, ST353
Vargas2022	Argentina	*K. pneumoniae*	OXA-48-like	*bla*OXA-48-like	NR	PCR	OXA-48-like + SHV-2 + CTX-M-15	Isolates Kp11, Kp9A, Kp13; ST17 (cluster A); OXA-48-like + SHV-2 + CTX-M-15 co-carriage
Barroso2023	Brazil	*K. pneumoniae*	KPC	*bla*KPC-2	NR	PCR, Sequencing	*bla*CTX-M-15 co-harbored in majority of isolates	ICU isolates only (C-ICU=7, G-ICU=8, N-ICU=5); all CR-Kp harbored *bla*KPC-2; NDM, OXA-48, VIM, IMP negative; pediatric population <15 years
Borelli2021	Brazil	*K. pneumoniae*	KPC	*bla*KPC-2	NR	WGS	NR	Strains 98M3 and 125M3 carry *bla*KPC-2 on identical IncX3 plasmids (~42kb); functional validation positive; gene flanked by Tn3 transposases
Borelli2021	Brazil	*K. pneumoniae*	CTX-M	*bla*CTX-M-15	NR	WGS	With blaOXA-1 in some strains	Reported in *K. pneumoniae* 508B; functional validation positive (8 clones); co-harbored with *bla*OXA-1 in some strains
Borelli2021	Brazil	*K. pneumoniae*	OXA	*bla*OXA-1	NR	WGS	Shared among *E. coli*, *K. pneumoniae*, and M. morganii (100% aa identity)	Gene shared among *E. coli*, *K. pneumoniae*, and *M. morganii* (100% aa identity) suggests horizontal transfer; functional validation positive
Borelli2021	Brazil	*K. pneumoniae*	LAP	*bla*LAP-2	NR	WGS	With qnrS1 on plasmid	Located on plasmid pKP508BN34 (~63kb) IncFII type; functional validation positive (2 clones); first report of this plasmid in Brazil, related to Asian strains
Lima2020	Brazil	*K. pneumoniae*	KPC	*bla*KPC-2	NR	Multiplex PCR	1 isolate with KPC-2 + OXA-48-like co-carriage	Prevalence not explicitly reported by species; 1 isolate with KPC-2 + OXA-48-like co-carriage
Lima2020	Brazil	*K. pneumoniae*	OXA-48-like	*bla*OXA-48-like	NR	Multiplex PCR	Co-carriage with blaKPC-2	Single isolate with co-carriage detected

Lorenzoni2017	Brazil	*K. pneumoniae*	KPC	*bla*KPC	NR	PCR	NR	Adult ICU; manual count from Table 2; additional Cardiac ICU: 2/2 *bla*KPC+; OXA-48, NDM, GES genes not detected
OchoaDiaz2022	Colombia	*K. pneumoniae*	KPC	*bla*KPC	32.50%	PCR	NR	Denominator is total KPN isolates, not only CR
Mejia-Limones2024	Ecuador	*K. pneumoniae*	KPC	*bla*KPC-2	35.70%	WGS	NR	Denominator includes all WGS-analyzed isolates (not exclusively CR); 5 Group C isolates with *bla*KPC-2; ST629 predominant among KPC-positive
Mejia-Limones2024	Ecuador	*K. pneumoniae*	KPC	*bla*KPC-3	NR	WGS	NR	FIRST REPORT of *bla*KPC-3 in Ecuador; isolate KPEC34K, ST45-2LV; associated with plasmid FII(pBK30683)
Soria-Segarra2020	Ecuador	*K. pneumoniae*	KPC	*bla*KPC-2	100.00%	Multiplex PCR	NR	*bla*KPC-2 confirmed in ST258, ST512, ST45; 22 STs identified by MLST; ST258 predominant (n=5); clonal transmission documented by PFGE (clusters A and B = 78.7%)
Garcia2024	Peru	*K. pneumoniae*	KPC	*bla*KPC	NR	Immunochromatography	No co-carriage detected	100% KPC in KPN-UCI; RESISIT-3 OKN K-SET detects protein not gene
GarciaCedron2023	Peru	*K. pneumoniae*	KPC	*bla*KPC	NR	Immunochromatography	NR	100% KPC in KPN; RESIST-3 O.K.N K-SET detects KPC, NDM, OXA-48
Vargas2022	Argentina	*A. baumannii*	IMP	*bla*IMP-1	NR	PCR	NR	Predominant in Audit 1 (2018); Cluster A (pulsotypes i, ii, iii)
Vargas2022	Argentina	*A. baumannii*	OXA-48-like	*bla*OXA-48-like	NR	PCR	Coexistence with *K. pneumoniae* OXA-48-like and E. coli OXA-48-like in 1 patient	Isolates Ab7 and Ab8; detected in V.A.E.1 and V.A.E.2; coexistence with *K. pneumoniae* and *E. coli* OXA-48-like in 1 patient
Vargas2022	Argentina	*A. baumannii*	VIM	*bla*VIM-1	NR	PCR	NR	Isolate Ab9, V.A.E.2, cluster B
Chagas2025	Brazil	*A. baumannii*	OXA-23-like	*bla*OXA-23	NR	RT-PCR	NR	*bla*OXA-23 only tested in *A. baumannii* per methods; 15/16 isolates positive
Lima2020	Brazil	*A. baumannii*	OXA-23-like	*bla*OXA-23-like	72.00%	Multiplex PCR	Co-carriage with blaOXA-51-like (intrinsic) in all isolates	Denominator 18 per figure legend; *bla*OXA-51-like (intrinsic) co-carried in all isolates; not extracted as acquired resistance gene
CastilloBejarano2023	Mexico	*A. baumannii*	OXA-24-like	*bla*OXA-24	100.00%	PCR	12 isolates co-carrying blaOXA-24 + *bla*IMP	Denominator includes 2 non-ICU isolates (9.5%); all 21 isolates harbored *bla*OXA-24
CastilloBejarano2023	Mexico	*A. baumannii*	IMP	*bla*IMP	57.00%	PCR	All *bla*IMP+ also carried blaOXA-24	Denominator includes 2 non-ICU isolates; 5/12 *bla*IMP detections occurred Aug-Oct 2020 (possible cluster)
SanchezUrtaza2025	Paraguay	*A. baumannii*	OXA-23-like	*bla*OXA-23	100.00%	PCR, WGS	NR	7 IC2 isolates with 2 copies of blaOXA-23 in Tn2006; 1 IC5 isolate with *bla*OXA-23 in Tn2008; selected sample (first isolate per ICU)
GarciaCedron2023	Peru	*A. baumannii*	NDM	*bla*NDM	NR	Immunochromatography	NR	NDM predominant in ABA (6/7)

GarciaCedron2023	Peru	*A. baumannii*	OXA-48	*bla*OXA-48	NR	Immunochromatography	NR	OXA-48 minority in ABA
Vargas2022	Argentina	*P. aeruginosa*	VIM	*bla*VIM-2	NR	PCR	Coexistence with A. baumannii IMP-1 in same patient	Single P. aeruginosa isolate reported; Audit 1; coexistence with A. baumannii IMP-1 in same patient
Lima2020	Brazil	*P. aeruginosa*	KPC	*bla*KPC-2	NR	Multiplex PCR	NR	Data extracted from graph; 2 isolates with *bla*KPC-2 per yellow bars
Souza2021	Brazil	*P. aeruginosa*	KPC	*bla*KPC-2	11.00%	PCR, DNA sequencing	NR	Only carbapenemase detected; sequencing confirmed KPC-2 variant
Souza2021	Brazil	*P. aeruginosa*	VIM	*bla*VIM	0.00%	PCR	NA	*bla*VIM-1 not detected
Souza2021	Brazil	*P. aeruginosa*	IMP	*bla*IMP	0.00%	PCR	NA	*bla*IMP-1 not detected
Souza2021	Brazil	*P. aeruginosa*	NDM	blaNDM	0.00%	PCR	NA	blaNDM-1 not detected
Souza2021	Brazil	*P. aeruginosa*	OXA-48-like	*bla*OXA-48	0.00%	PCR	NA	*bla*OXA-48 not detected
Souza2021	Brazil	*P. aeruginosa*	SPM	*bla*SPM	0.00%	PCR	NA	*bla*SPM not detected (MBL endemic in Brazil)
Souza2021	Brazil	*P. aeruginosa*	GIM	*bla*GIM	0.00%	PCR	NA	blaGIM not detected
OchoaDiaz2022	Colombia	*P. aeruginosa*	KPC	*bla*KPC	5.70%	PCR	NR	Denominator is total PAE isolates, not only CR; tested in all HAI isolates
OchoaDiaz2022	Colombia	*P. aeruginosa*	OXA-48-like	*bla*OXA-48	1.80%	PCR	NR	Denominator is total PAE isolates; OXA-48 identified exclusively in PAE
OchoaDiaz2022	Colombia	*P. aeruginosa*	OXA-23	*bla*OXA-23	8.80%	PCR	NR	Denominator is total PAE isolates; unusual finding - OXA-23 typically in Acinetobacter
GarciaCedron2023	Peru	*P. aeruginosa*	NDM	*bla*NDM	NR	Immunochromatography	NR	NDM predominant in PAE (11/16)
GarciaCedron2023	Peru	*P. aeruginosa*	OXA-48	*bla*OXA-48	NR	Immunochromatography	NR	OXA-48 second most frequent in PAE
GarciaCedron2023	Peru	*P. aeruginosa*	KPC	*bla*KPC	NR	Immunochromatography	NR	KPC rare in PAE (1/16)
Vargas2022	Argentina	*E. coli*	OXA-48-like	*bla*OXA-48-like	NR	PCR	Coexistence with *K. pneumoniae* OXA-48-like and *A. baumannii* OXA-48-like (1 patient); single isolate in another patient	V.A.E.1: 1 patient with triple colonization (*K. pneumoniae, A. baumannii, E. coli* OXA-48-like); V.A.E.2: 1 patient with E. coli OXA-48-like
Borelli2021	Brazil	*E. coli*	KPC	*bla*KPC-2	NR	WGS	NR	Strain 126M3; gene identical to *K. pneumoniae* (100% identity); plasmid localization not achieved due to assembly limitations; functional validation positive (28 clones)
Lima2020	Brazil	*E. coli*	KPC	*bla*KPC-2	NR	Multiplex PCR	NR	Single *E. coli* CR isolate tested

OchoaDiaz2022	Colombia	*E. coli*	KPC	*bla*KPC	5.10%	PCR	NR	Denominator is total ECO isolates, not only CR
OchoaDiaz2022	Colombia	*E. coli*	OXA-23	*bla*OXA-23	2.60%	PCR	NR	Denominator is total ECO isolates; unusual finding - OXA-23 typically in Acinetobacter
Soria-Segarra2020	Ecuador	*E. coli*	KPC	*bla*KPC	100.00%	Multiplex PCR	NR	Specific KPC variant not reported for this species
Garcia2024	Peru	*E. coli*	NDM	*bla*NDM	NR	Immunochromatography	No co-carriage detected	RESISIT-3 OKN K-SET test
Garcia2024	Peru	*E. coli*	OXA-48-like	*bla*OXA-48	NR	Immunochromatography	No co-carriage detected	RESISIT-3 OKN K-SET test
GarciaCedron2023	Peru	*E. coli*	NDM	*bla*NDM	NR	Immunochromatography	NR	NDM predominant in ECO (5/7)
GarciaCedron2023	Peru	*E. coli*	OXA-48	*bla*OXA-48	NR	Immunochromatography	NR	OXA-48 minority in ECO
Soria-Segarra2020	Ecuador	*Enterobacter cloacae* complex	KPC	*bla*KPC	100.00%	Multiplex PCR	NR	Specific KPC variant not reported for this species
Garcia2024	Peru	*Enterobacter cloacae* complex	NDM	*bla*NDM	NR	Immunochromatography	No co-carriage detected	Very low N (n=1)
GarciaCedron2023	Peru	*Enterobacter cloacae* complex	NDM	*bla*NDM	NR	Immunochromatography	NR	Very low N (n=1); interpret with caution

Table S9. Search strategies.

Search strategy	Database
("Carbapenem-Resistant Enterobacteriaceae"[Mesh] OR "Carbapenem-Resistant Enterobacteriaceae"[tiab] OR "carbapenem resistance"[tiab] OR "carbapenem resistant"[tiab] OR "carbapenem-resistant"[tiab] OR "carbapenemase-producing"[tiab] OR carbapenemase*[tiab] OR CRE[tiab] OR CR-GNB[tiab] OR CRGN[tiab] OR "metallo-beta-lactamase"[tiab] OR MBL[tiab] OR KPC[tiab] OR "blaKPC"[tiab] OR NDM[tiab] OR "blaNDM"[tiab] OR OXA[tiab] OR "blaOXA"[tiab] OR VIM[tiab] OR IMP[tiab] OR "beta-Lactamases"[Mesh])<br>AND<br>("Intensive Care Units"[Mesh] OR "Critical Care"[Mesh] OR "intensive care"[tiab] OR "critical care"[tiab] OR ICU[tiab] OR ICUs[tiab] OR PICU[tiab] OR NICU[tiab] OR "burn unit"[tiab] OR "unidad de cuidados intensivos"[tiab] OR "unidade de terapia intensiva"[tiab] OR UTI[tiab] OR UCI[tiab])<br>AND<br>("Latin America"[Mesh] OR "South America"[Mesh] OR "Caribbean Region"[Mesh] OR "Central America"[Mesh] OR "Latin America"[tiab] OR "South America"[tiab] OR Caribbean[tiab] OR "Central America"[tiab] OR Argentina[tiab] OR Bolivia[tiab] OR Brazil[tiab] OR Brasil[tiab] OR Chile[tiab] OR Colombia[tiab] OR "Costa Rica"[tiab] OR Cuba[tiab] OR "Dominican Republic"[tiab] OR Ecuador[tiab] OR "El Salvador"[tiab] OR Guatemala[tiab] OR Honduras[tiab] OR Mexico[tiab] OR Nicaragua[tiab] OR Panama[tiab] OR Paraguay[tiab] OR Peru[tiab] OR Uruguay[tiab] OR Venezuela[tiab] OR "West Indies"[tiab] OR Bahamas[tiab] OR Barbados[tiab] OR Belize[tiab] OR Guyana[tiab] OR Haiti[tiab] OR Jamaica[tiab] OR Suriname[tiab] OR "Trinidad and Tobago"[tiab])<br>AND<br>("Gram-Negative Bacteria"[Mesh] OR "Enterobacteriaceae"[Mesh] OR "Klebsiella pneumoniae"[Mesh] OR "Acinetobacter baumannii"[Mesh] OR "Pseudomonas aeruginosa"[Mesh] OR "Escherichia coli"[Mesh] OR "gram-negative"[tiab] OR "gram negative"[tiab] OR GNB[tiab] OR Enterobacteriaceae[tiab] OR Enterobacterales[tiab] OR "Klebsiella"[tiab] OR "Acinetobacter"[tiab] OR "Pseudomonas"[tiab] OR "Enterobacter"[tiab] OR "Escherichia coli"[tiab] OR "E. coli"[tiab])<br>AND<br>(("2015/01/01"[Date - Publication] : "2025/12/31"[Date - Publication]))	**Pubmed**
TITLE-ABS-KEY ("carbapenem resistance" OR "carbapenem-resistant" OR "carbapenem resistant" OR "carbapenemase-producing" OR carbapenemase* OR cre OR "cr-gnb" OR crgn OR kpc OR "blakpc" OR ndm OR "blandm" OR oxa OR "blaoxa" OR vim OR imp OR "metallo-beta-lactamase" OR mbl) AND TITLE-ABS-KEY ("intensive care" OR "critical care" OR icu OR icus OR picu OR nicu OR "burn unit" OR "unidad de cuidados intensivos" OR "unidade de terapia intensiva" OR uti OR uci) AND TITLE-ABS-KEY ("Latin America" OR	**Scopus**

"South America" OR caribbean OR "Central America" OR argentina OR bolivia OR brazil OR brasil OR chile OR colombia OR "Costa Rica" OR cuba OR "Dominican Republic" OR ecuador OR "El Salvador" OR guatemala OR honduras OR mexico OR nicaragua OR panama OR paraguay OR peru OR uruguay OR venezuela OR bahamas OR barbados OR belize OR guyana OR haiti OR jamaica OR suriname OR "Trinidad and Tobago") AND TITLE-ABS-KEY ("gram-negative" OR "gram negative" OR gnb OR enterobacteriaceae OR enterobacterales OR klebsiella OR acinetobacter OR pseudomonas OR enterobacter OR "Escherichia coli" OR "E. coli") AND PUBYEAR > 2014 AND PUBYEAR < 2026	
(tw:(("carbapenem resistance" OR "resistencia a carbapenem" OR "resistencia a carbapenemicos" OR "resistência a carbapenem" OR "carbapenem-resistant" OR "resistente a carbapenem" OR "carbapenemase" OR "carbapenemasa" OR CRE OR KPC OR NDM OR OXA OR VIM OR IMP OR "metallo-beta-lactamase" OR "metalo-beta-lactamasa" OR MBL)))<br>AND<br>(tw:(("intensive care" OR "critical care" OR "cuidados intensivos" OR "terapia intensiva" OR "cuidados criticos" OR ICU OR PICU OR NICU OR UTI OR UCI)))<br>AND<br>(tw:(("Latin America" OR "America Latina" OR "South America" OR "America del Sur" OR "America do Sul" OR Caribbean OR Caribe OR "Central America" OR "America Central" OR Argentina OR Bolivia OR Brazil OR Brasil OR Chile OR Colombia OR "Costa Rica" OR Cuba OR "Dominicana" OR Ecuador OR "El Salvador" OR Guatemala OR Honduras OR Mexico OR Nicaragua OR Panama OR Paraguay OR Peru OR Uruguay OR Venezuela)))<br>AND<br>(tw:(("gram-negative" OR "gram negativos" OR "gram-negativos" OR GNB OR Enterobacteriaceae OR Enterobacteriaceas OR Enterobacterales OR Klebsiella OR Acinetobacter OR Pseudomonas OR Enterobacter OR "Escherichia coli" OR "E. coli")))<br>AND (year_cluster:[2015 TO 2025])	**Lilacs/SciELO**

Supplementary figures

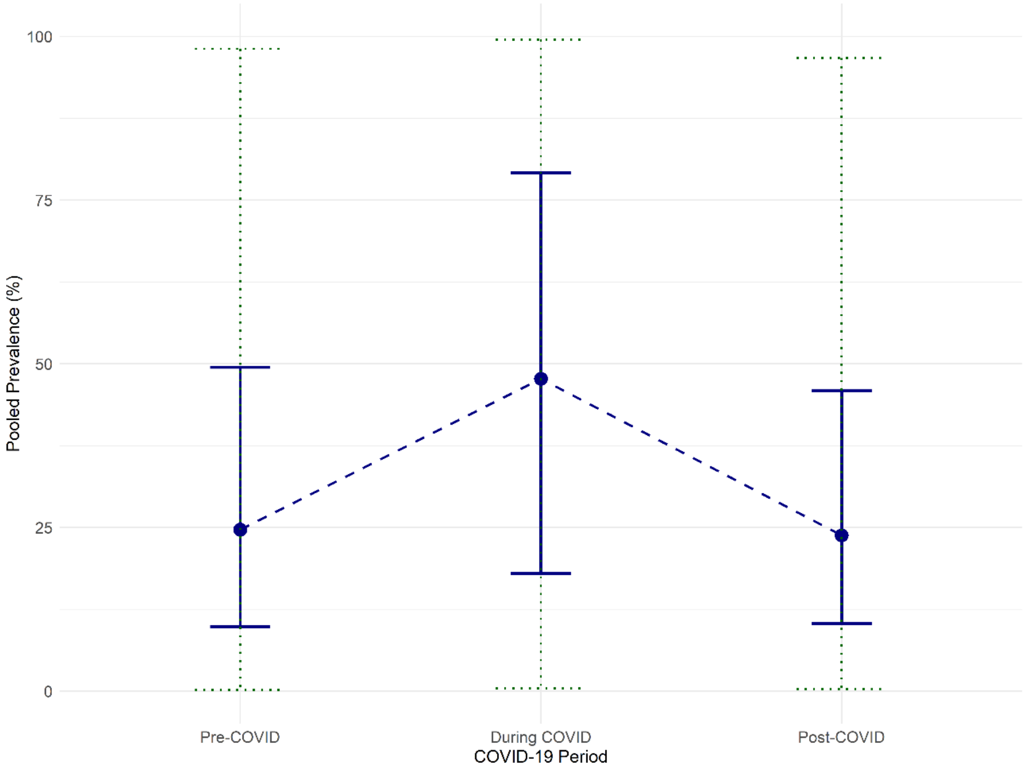

Figure S1. Pooled carbapenem resistance prevalence with 95% confidence and prediction intervals stratified by COVID-19 pandemic period.

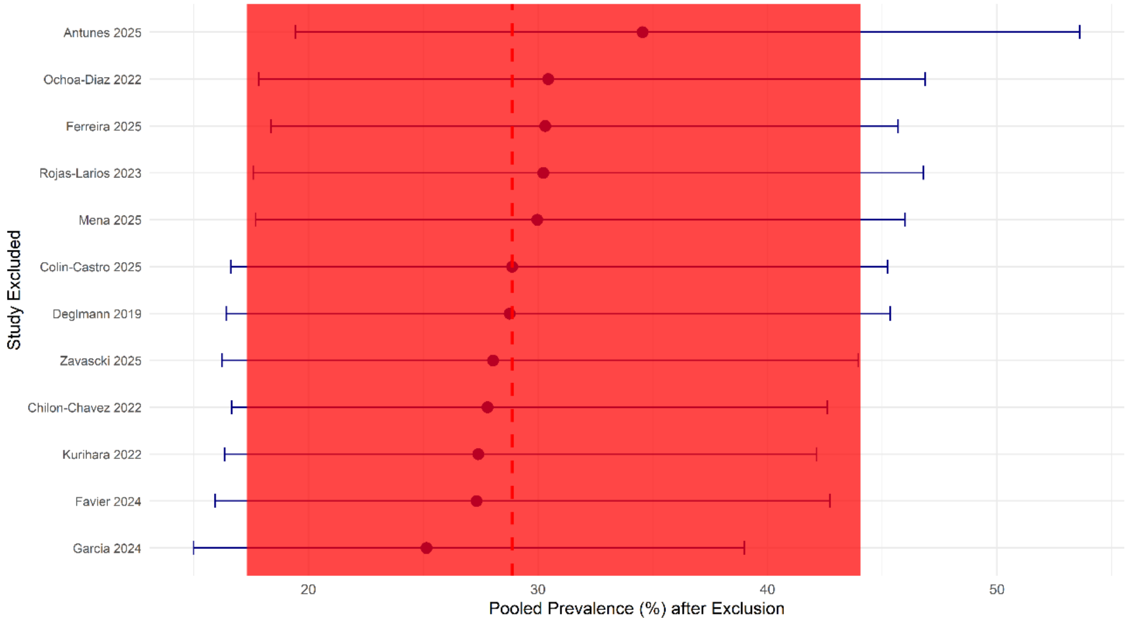

Figure S2. Leave-one-out sensitivity analysis for the overall pooled carbapenem resistance prevalence estimate.

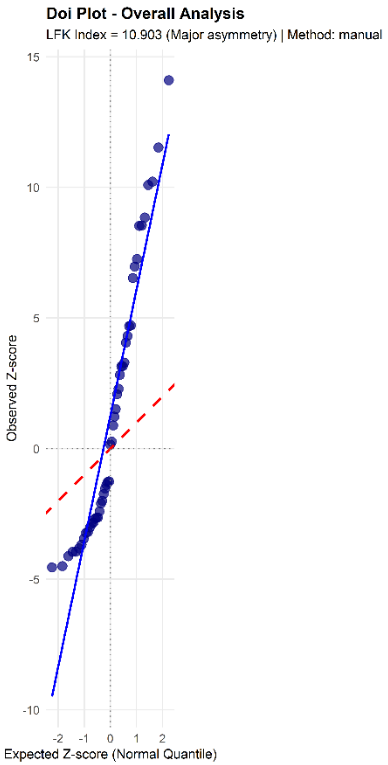

Figure S3. Doi plot for publication bias assessment in the overall meta-analysis (LFK index = 10.90).

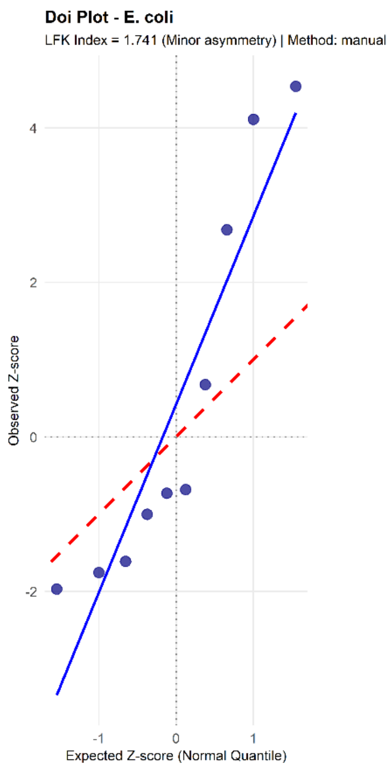

Figure S4. Doi plot for publication bias assessment in *Escherichia coli* subgroup analysis (LFK index = 1.74).

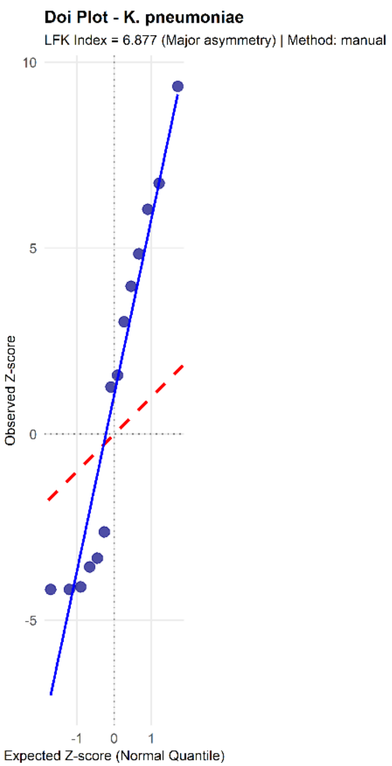

Figure S5. Doi plot for publication bias assessment in *Klebsiella pneumoniae* subgroup analysis (LFK index = 6.88).

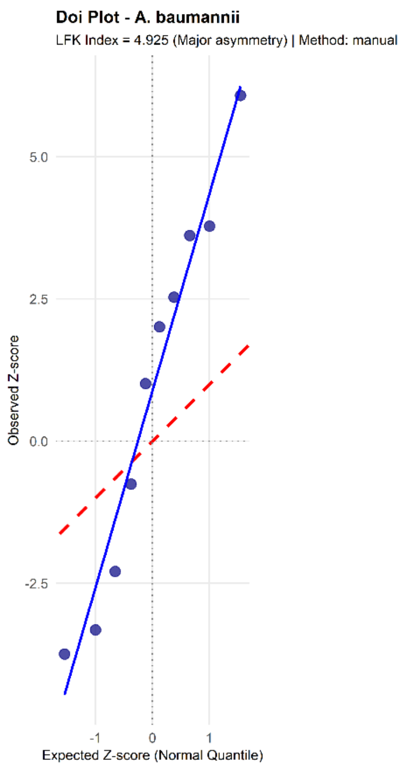

Figure S6. Doi plot for publication bias assessment in *Acinetobacter baumannii* subgroup analysis (LFK index = 4.93).

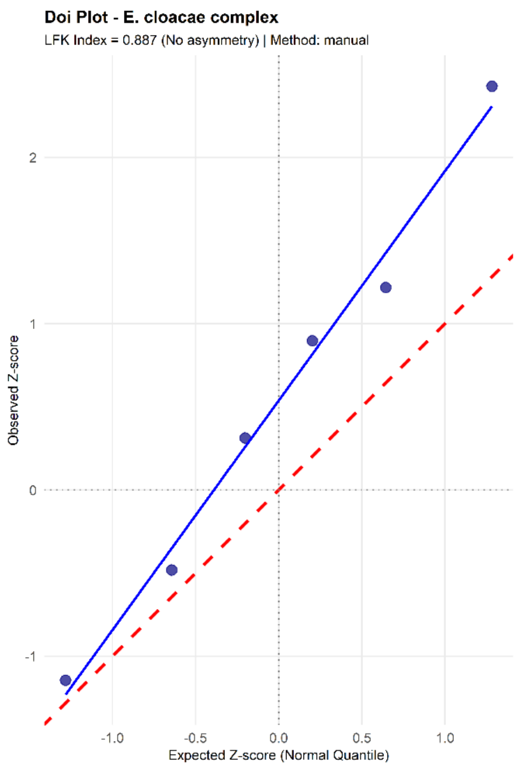

Figure S7. Doi plot for publication bias assessment in *Enterobacter cloacae* complex subgroup analysis (LFK index = 0.89).

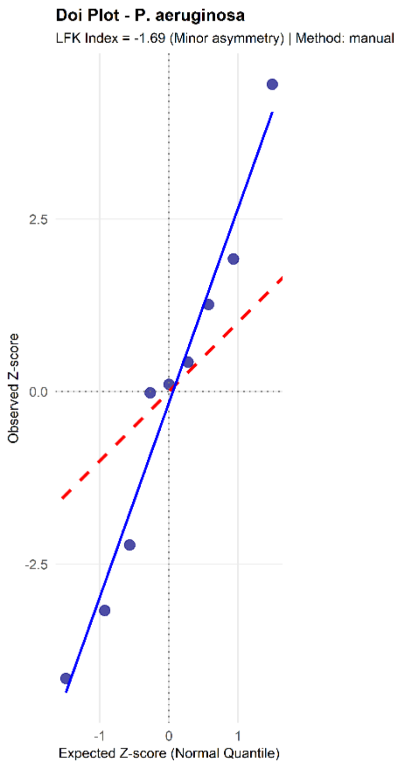

Figure S8. Doi plot for publication bias assessment in *Pseudomonas aeruginosa* subgroup analysis (LFK index = −1.89).
